# Use of a Bacterial Artificial Chromosome to Generate Recombinant SARS-CoV-2 Expressing Robust Levels of Reporter Genes

**DOI:** 10.1128/spectrum.02732-22

**Published:** 2022-11-07

**Authors:** Chengjin Ye, Luis Martinez-Sobrido

**Affiliations:** a Texas Biomedical Research Institute, San Antonio, Texas, USA; University of Georgia

**Keywords:** BAC, COVID-19, SARS-CoV-2, bacterial artificial chromosome, coronavirus, pBeloBAC, recombinant virus, reporter genes, reverse genetics

## Abstract

Reporter-expressing recombinant virus represents an excellent option and a powerful tool to investigate, among others, viral infection, pathogenicity, and transmission, as well as to identify therapeutic compounds that inhibit viral infection and prophylactic vaccines. To combat the ongoing coronavirus disease 2019 (COVID-19) pandemic, we have established a robust bacterial artificial chromosome (BAC)-based reverse genetics (RG) system to rapidly generate recombinant severe acute respiratory syndrome coronavirus 2 (rSARS-CoV-2) to study the contribution of viral proteins in viral pathogenesis. In addition, we have engineered reporter-expressing recombinant viruses in which we placed the reporter genes upstream of the viral nucleocapsid (N) gene to promote high levels of reporter gene expression, which facilitates the study of SARS-CoV-2 *in vitro* and *in vivo*. To date, we have shared our BAC-based RG system with more than 100 laboratories around the world, which has helped to expedite investigations with SARS-CoV-2. However, genetic manipulation of the BAC containing the entire SARS-CoV-2 genome (~30,000 nt) is challenging. Herein, we provide the technical details to engineer rSARS-CoV-2 using the BAC-based RG approach. We describe (i) assembly of the full-length (FL) SARS-CoV-2 genome sequences into the empty pBeloBAC, (ii) verification of pBeloBAC-FL, (iii) cloning of a Venus reporter gene into pBeloBAC-FL, and (iv) recovery of the Venus-expressing rSARS-CoV-2. By following this protocol, researchers with knowledge of basic molecular biology and gene engineering techniques will be able to generate wild-type (WT) and reporter-expressing rSARS-CoV-2.

**IMPORTANCE** We have established a bacterial artificial chromosome (BAC)-based RG system to generate recombinant severe acute respiratory syndrome coronavirus 2 (rSARS-CoV-2) and to engineer reporter-expressing recombinant viruses to assess viral infection *in vitro* and *in vivo*. To date, we have shared our BAC-based RG system with more than 100 laboratories around the world, which has helped to expedite investigations with SARS-CoV-2. However, genetic manipulation of the BAC containing the full-length SARS-CoV-2 genome of ~30,000 nucleotides is challenging. Here, we provide all the detailed experimental steps required for the successful generation of wild-type (WT) recombinant SARS-CoV-2 (rSARS-CoV-2). Likewise, we provide a comprehensive protocol on how to generate and rescue rSARS-CoV-2 expressing high levels of a Venus fluorescent reporter gene from the locus of the viral nucleocapsid (N) protein. By following these protocols, researchers with basic knowledge in molecular biology will be able to generate WT and Venus-expressing rSARS-CoV-2 within 40 days.

## INTRODUCTION

Coronaviruses (CoVs) are enveloped, single-stranded, positive-sense RNA viruses belonging to the order *Nidovirales* that are responsible for causing seasonal mild respiratory illness (e.g., 229E, NL63, OC43, and HKU1) or fatal disease (e.g., severe acute respiratory syndrome coronavirus [SARS-CoV] and Middle East respiratory syndrome coronavirus [MERS-CoV]) in humans (1–3). The emergence of SARS-CoV-2 in December of 2019 initiated a worldwide pandemic of CoV disease 2019 (COVID-19) that is still threatening global public health (4–7). To investigate this newly emerged virus, we have established a robust bacterial artificial chromosome (BAC)-based reverse genetics (RG) system for SARS-CoV-2 (8). By using this system, we have engineered recombinant SARS-CoV-2 (rSARS-CoV-2) expressing fluorescent (mCherry or Venus) or bioluminescent (nanoluciferase [Nluc]) reporter genes that can be used for the screening of compounds with antiviral activity *in vitro* (9). However, this initial approach based on the substitution of the viral ORF7a with the reporter gene resulted in low levels of reporter gene expression, making it difficult to detect viral plaques with a ChemiDoc imaging system *in vitro* or viral replication in K18 human angiotensin-converting enzyme 2 (hACE2) transgenic mice using *in vivo* imaging systems (IVIS) (10). To increase the expression level of reporter genes, we developed a novel strategy in which reporter genes were placed upstream of the viral nucleocapsid (N) gene using the 2A autoproteolytic cleavage site of porcine teschovirus 1 (PTV1) (10). The expression level of reporter genes was dramatically increased with the new reporter rSARS-CoV-2, allowing the detection of viral infection using a ChemiDoc imaging system or IVIS (10). Collectively, this approach offers researchers a BAC-based RG approach to generate rSARS-CoV-2 expressing robust levels of reporter genes without deleting any viral genes.

Two reports initially described the assembly of the entire SARS-CoV-2 genome by *in vitro* ligation (11) or homologous recombination in yeast (12). However, both approaches rely on the *in vitro* transcription of the viral genome using a bacteriophage T7 promoter, a process that requires careful control of experimental variables and extraordinary technical expertise and is therefore restricted to only a few laboratories. To overcome this limitation, we developed a BAC-based RG system for SARS-CoV-2 (8). It applies the principles previously established to develop RG for other CoVs (13–15) and other single-stranded positive-sense RNA viruses (16, 17). After analyzing the complete genome sequences of SARS-CoV-2 and selecting appropriate restriction sites, we designed and chemically synthesized a set of five fragments (F1 to F5) covering the entire viral genome (8). The F1 segment contains a cytomegalovirus (CMV) immediate early promoter, the first 673 nucleotides of the SARS-CoV-2 genome, unique multiple-cloning sites selected for the assembly of the viral genome segments F2 to F5, the last 4,585 nucleotides of the SARS-CoV-2 genome, the hepatitis delta virus ribozyme (Rz), and the bovine growth hormone (bGH) termination and polyadenylation sequences. The SARS-CoV-2 F1 segment was first synthesized and cloned into the empty pBeloBAC. Then, the segments F2 to F5 were sequentially cloned into the plasmid using the unique multiple-cloning restriction sites introduced into F1. This method has been described previously by our laboratory, showing that the BAC RG approach gives rapid recombinant virus rescue by using a simple and conventional cell transfection technique (8). With this method, we have also generated a stable rSARS-CoV-2 expressing robust levels of reporter genes (10).

However, because the pBeloBAC plasmid that harbors all elements required for efficient viral rescue and the SARS-CoV-2 genome is ~38,000 bp long, it is challenging to manipulate using conventional molecular biology methods and techniques. To assist other researchers to overcome challenges with the use of the SARS-CoV-2 BAC-based RG system, we provide here all the technical information and detailed protocols necessary for the assembly of the entire SARS-CoV-2 genome into pBeloBAC and its manipulation to generate rSARS-CoV-2 expressing robust levels of the reporter gene Venus. Importantly, this protocol could be employed by other researchers to generate wild-type (WT) SARS-CoV-2, rSARS-CoV-2 expressing other reporters, and/or viral mutants of interest by following or adapting the described experimental procedures.

## RESULTS AND DISCUSSION

**FIG 1:**
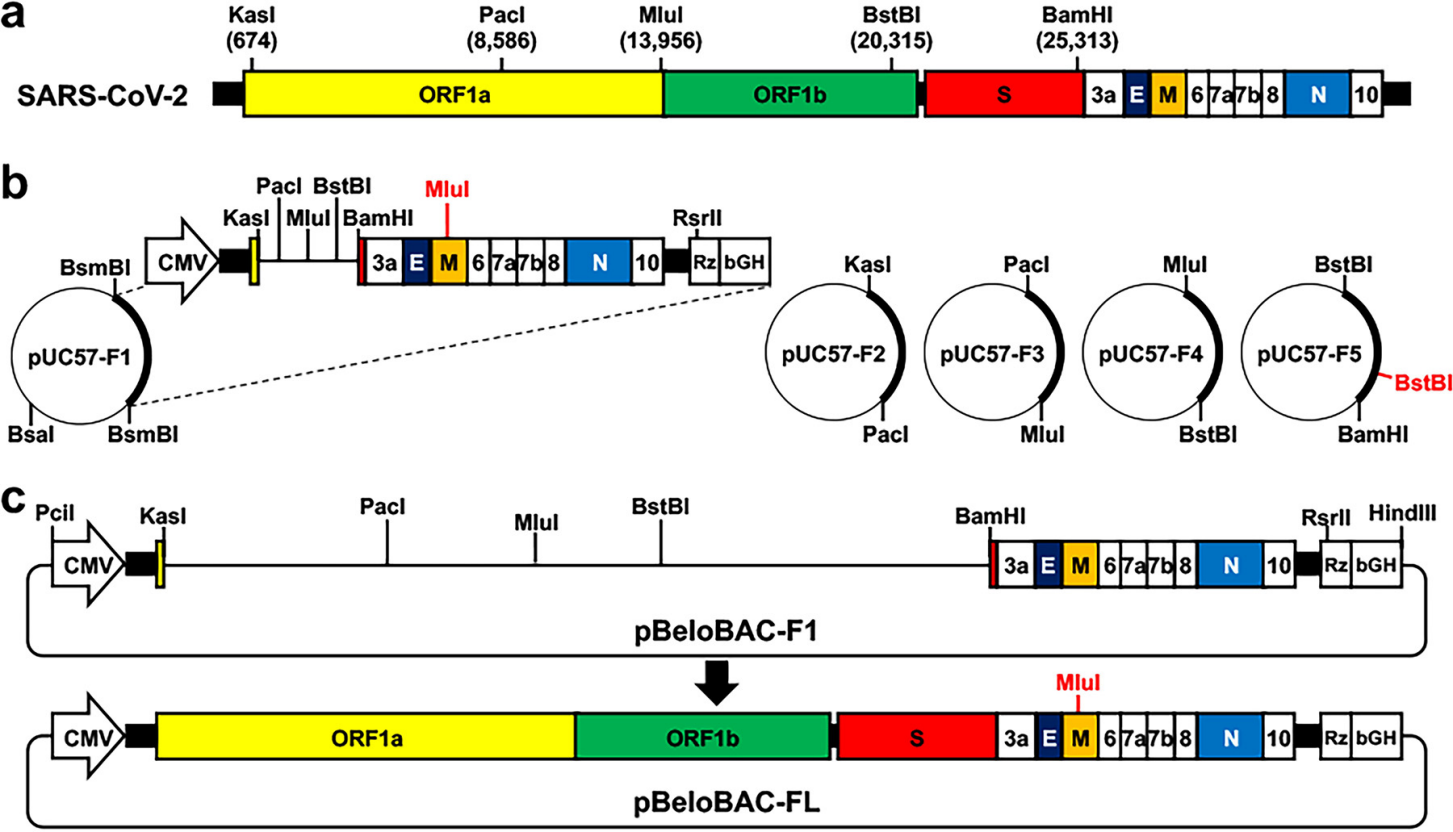
Overview of a BAC-based RG system for generation of rSARS-CoV-2. (a) Schematic representation of the SARS-CoV-2 genome. Unique restriction sites used for viral genome assembly are indicated. (b) Commercially synthesized fragments (F1 to F5) in pUC57 plasmids. Restriction sites used to release each of the viral fragments are indicated. The MluI and BstBI sites in red are the restriction sites that have been removed by silent mutation and used as genetics tags. (c) Assembly of the entire SARS-CoV-2 genome into the empty pBeloBAC to generate the full-length rescue plasmid (pBeloBAC-FL).

**FIG 2:**
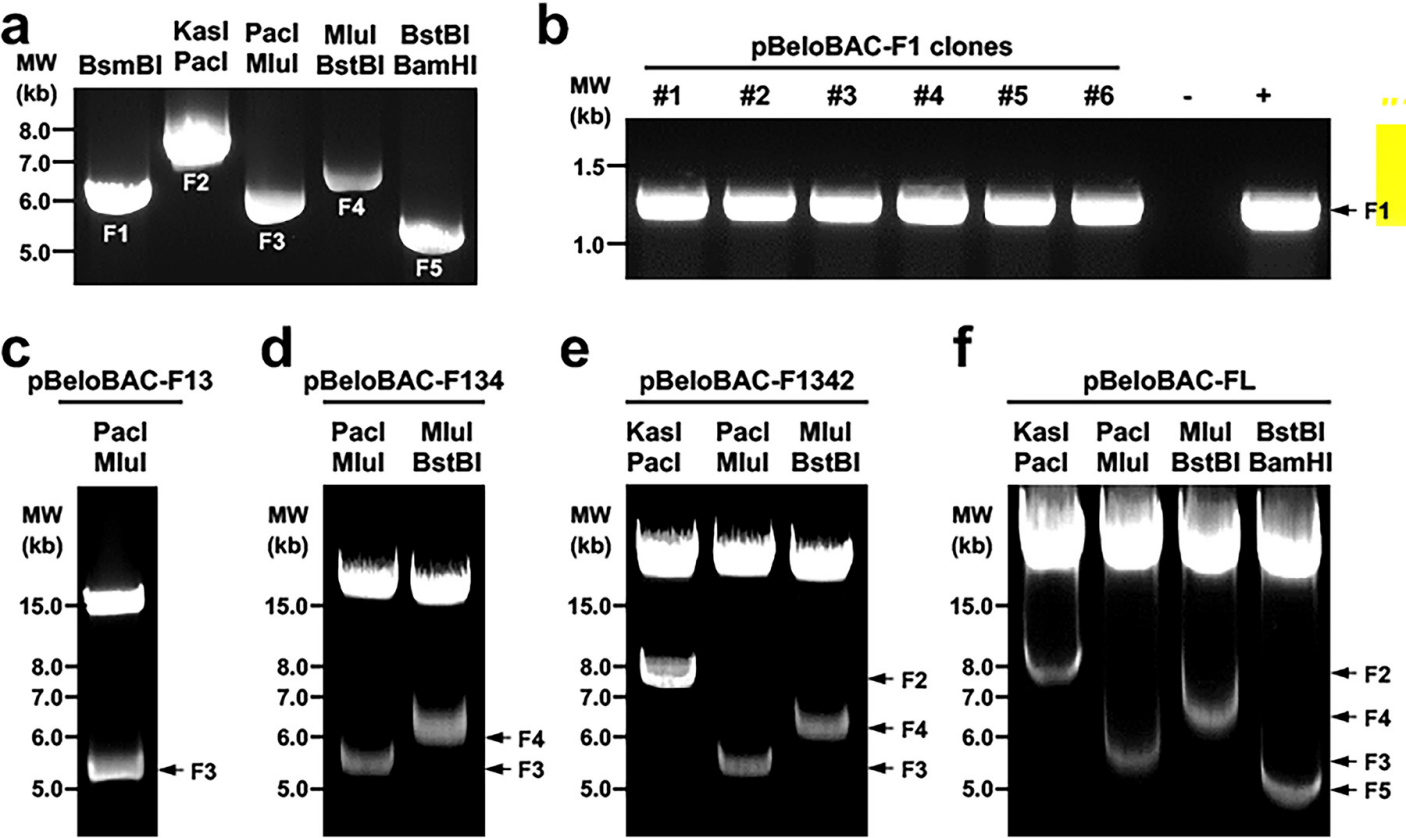
Assembly of the entire SARS-CoV-2 genome into pBeloBAC. (a) Fragments (F1 to F5) released from pUC57 plasmids. (b) PCR screening for colonies positive for pBeloBAC-F1. (c) Restriction digestion analysis of pBeloBAC-F13 using PacI and MluI. (d) Restriction digestion analysis of pBeloBAC-F134 using PacI and MluI (F3) and MluI and BstBI (F4). (e) Restriction digestion analysis of pBeloBAC-F1342 using KasI and PacI (F2), PacI and MluI (F3), and MluI and BstBI (F4). (f) Restrict digestion confirmation of pBeloBAC-FL using KasI and PacI (F2), PacI and MluI (F3), MluI and BstBI (F4), and BstBI and BamHI (F5).

**FIG 3:**
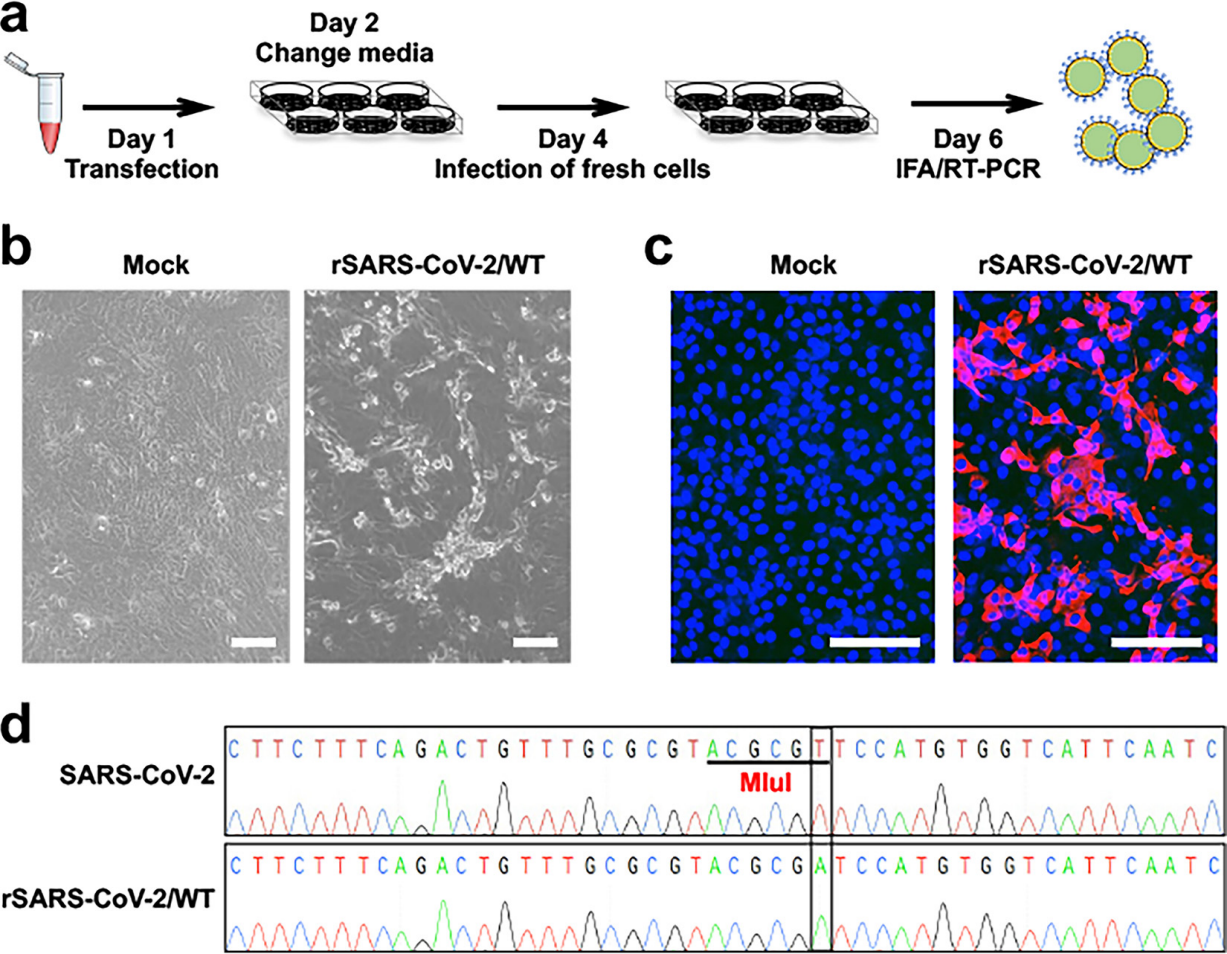
Recovery of rSARS-CoV-2 from pBeloBAC-FL. (a) Protocol used for recovery of rSARS-CoV-2 from pBeloBAC-FL using Lipofectamine 2000 transfection in Vero E6 cells. (b) CPE caused by rSARS-CoV-2/WT in Vero E6 cells. Bars, 100 μm. (c) Confirmation of the rescued rSARS-CoV-2/WT by IFA using a mouse antibody against viral N protein and a TRITC-labeled donkey anti-mouse IgG secondary antibody. The nucleus was stained with DAPI. (d) Sanger sequencing of the M gene of the natural isolate SARS-CoV-2 (top) and the recombinant SARS-CoV-2 (bottom) showed that the MluI site was removed by silent mutation.

**FIG 4:**
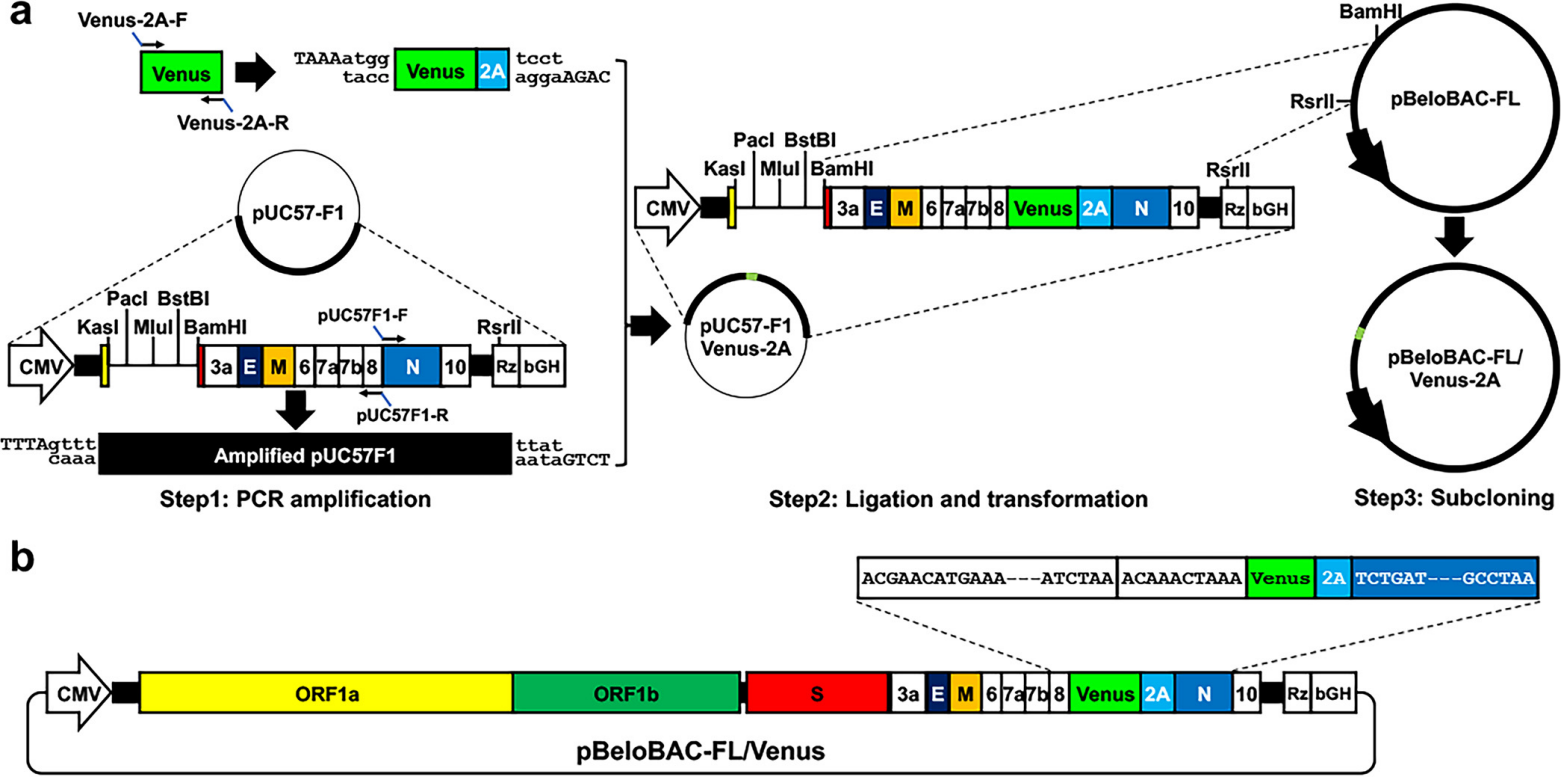
Overview of the experimental approach to generate rSARS-CoV-2 expressing Venus from the N protein locus. (a) Schematic representation of the experimental flow to assemble Venus into pUC57-FA and subclone it into pBeloBAC-FL. (b) Schematic depiction of pBeloBAC-FL/Venus-2A.

**FIG 5:**
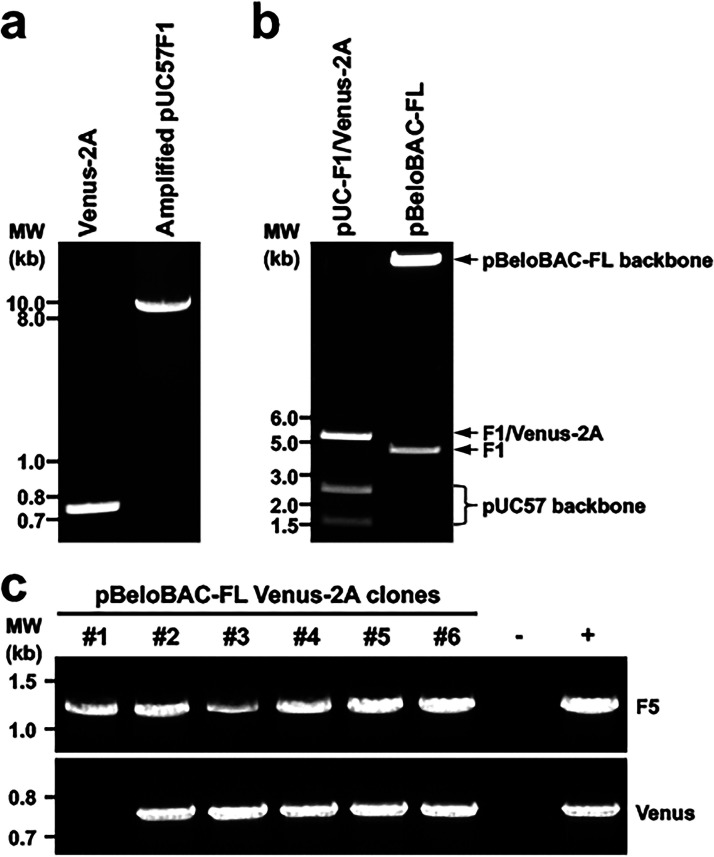
Assembly of F1 containing Venus-2A into pBeloBAC for generation of rSARS-CoV-2/Venus-2A. (a) PCR amplification of Venus-2A and pUC57F1. (b) Purification of the F1/Venus-2A released from pUC57-F1/Venus-2A and the pBeloBAC-FL backbone after BamHI and RsrII digestion to remove the parental F1 segment for subcloning of F1/Venus-2A. (c) PCR screening of pBeloBAC-FL/Venus-2A colonies.

**FIG 6:**
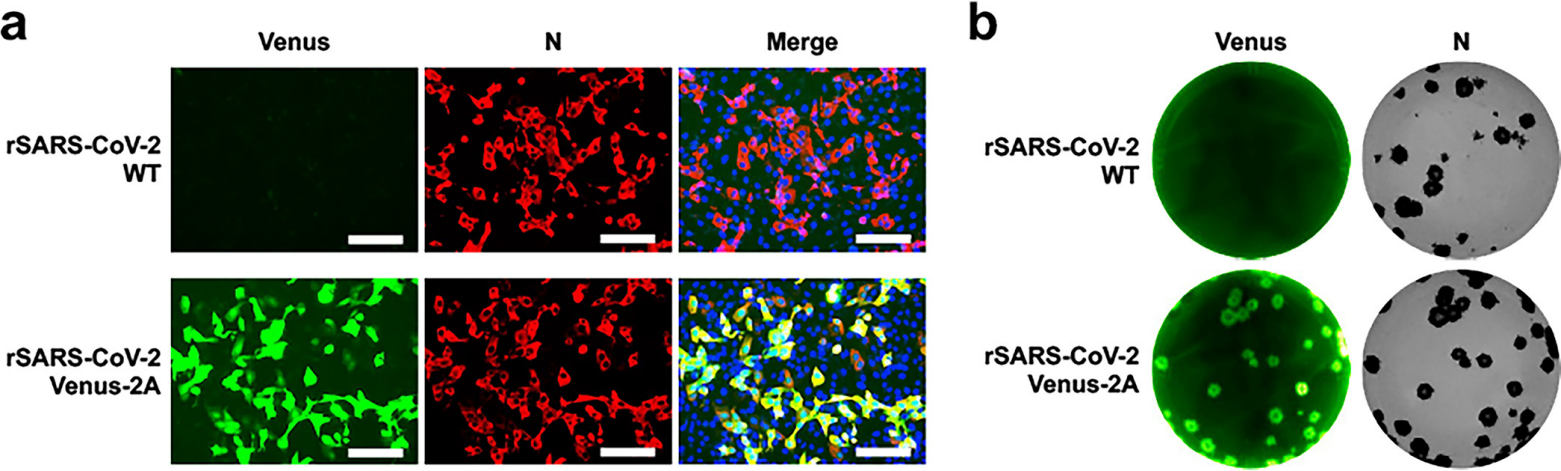
Characterization of rSARS-CoV-2/Venus-2A. (a) Fluorescent (Venus) and IFA (N) confirmation of rSARS-CoV-2/Venus-2A rescue. rSARS-CoV-2/WT was included as an internal control. (b) Detection of fluorescent plaques formed by rSARS-CoV-2/Venus-2A with a ChemiDoc imaging system. Immunostaining with the N protein 1C7C7 MAb of the plaques formed by rSARS-CoV-2/Venus-2A and rSARS-CoV-2/WT.

This protocol can be used to successfully assemble the entire SARS-CoV-2 genome into empty pBeloBAC (Fig. 1 and 2), resulting in efficient recovery of rSARS-CoV-2 or WT SARS-CoV-2, which exhibits a cytopathic effect (CPE) on Vero E6 cells infected with cell culture supernatants from transfected cells (Fig. 3). The rSARS-CoV-2/WT rescue can be further confirmed by immunofluorescent assay (IFA) using a monoclonal antibody (1C7C7) against the viral N protein (Fig. 3), and by Sanger sequencing the reverse transcription-PCR (RT-PCR) product of the M gene to demonstrate the recombinant nature of the rescued SARS-CoV-2 (Fig. 3). By using pBeloBAC-FL as a backbone, this protocol efficiently introduces the reporter gene Venus into pBeloBAC-FL (Fig. 4 and 5) and generates rSARS-CoV-2/Venus-2A expressing robust levels of Venus (Fig. 6), allowing the virus to be readily detected as fluorescent plaques under a ChemiDoc imaging system (Fig. 6).

RG techniques represent a powerful tool to answer important questions about viral infection, transmission, and pathogenesis. In addition, RG approaches have been used to develop attenuated forms of viruses that can be used as live-attenuated vaccines (LAV) for the prevention of viral infections. Likewise, similar RG systems have been used to genetically manipulate the viral genome to express foreign genes, including those encoding reporter fluorescent or luciferase proteins, in order to establish viral inhibition assays to facilitate the identification of biologicals affecting viral infection, including antivirals and antibodies (9).

The key features of this protocol are the assembly of large DNA fragments into the empty pBeloBAC plasmid and the genetic manipulation of pBeloBAC and its derivates. In this protocol, we describe the assembly of the entire viral genome of SARS-CoV-2 strain WA-1, one of the largest RNA viral genomes, into the empty pBeloBAC and the generation of rSARS-CoV-2 expressing robust levels of a fluorescent Venus reporter gene. Moreover, similar experimental procedures can be used to delete (18), replace (9), or mutate (19) SARS-CoV-2 genome sequences or to insert additional foreign genes. In addition, this protocol can be also applied to establish RG systems to generate recombinant forms of other nonsegmented positive-stranded RNA viruses, as well as viral genome sequences that are either unstable or unable to be genetically assembled and manipulated in other DNA vector backbones.

Our SARS-CoV-2 BAC-based RG system has several advantages over alternative methods. First, our RG approach based on the use of a BAC DNA does not rely on *in vitro* RNA transcription using a bacteriophage T7 promoter and RNA electroporation. Second, our BAC DNA transfection protocol based on the use of Lipofectamine 2000 is less technically challenging than electroporating cells with RNA molecules produced *in vitro* to recover rSARS-CoV-2. Third, once the entire SARS-CoV-2 genome sequences are successfully assembled into pBeloBAC, viral fragments do not need to be prepared each time. Fourth, the BAC DNA is highly stable, which allows sharing and transport between laboratories. Last, but not least, the levels of reporter gene expression using our innovative PTV1 2A approach from the locus of the viral N gene are dramatically higher than those obtained by expressing the reporter gene from the locus of the viral ORF7a (9). Importantly, our approach does not require replacing a viral gene with the reporter gene, in contrast to the previous strategy of expressing foreign genes by substituting them for the viral ORF7a gene (9, 10).

However, assembling the entire viral genome into the empty pBeloBAC for successful rescue of SARS-CoV-2 is time-consuming. To overcome this limitation, F2 to F5 could be assembled into pBeloBAC-F1 simultaneously, rather than stepwise. Since *in vitro* ligation of seven viral fragments has been described in the literature (20), simultaneous assembly of F2 to F5 into pBeloBAC-F1 would be feasible. Alternatively, assembly of F2 to F5 into pBeloBAC-F1 could be accomplished by using a yeast recombination-based system, as previously described (12). Another limitation of our RG system is that for successful results, it is important to obtain a yield of the pBeloBAC plasmid, which is maintained as a single copy in each bacterium. To obtain sufficient pBeloBAC plasmid for a one-time rescue by transfection into Vero E6 cells using Lipofectamine 2000, we suggest preparing the pBeloBAC plasmid from a minimum volume of 25 mL of overnight-cultured bacteria.

Our experimental design relies on the molecular genomic assembly of five fragments that span the entire SARS-CoV-2 genome into the empty pBeloBAC to establish an RG system for SARS-CoV-2. By using pBeloBAC-FL, we introduce a Venus reporter gene upstream of the N gene to generate a rSARS-CoV-2 expressing robust levels of Venus (rSARS-CoV-2/Venus-2A). While the five viral fragments were commercially synthesized, it is also feasible that they may be amplified from SARS-CoV-2-infected samples, or from purified SARS-CoV-2 RNA, using RT-PCR and then cloned into a plasmid vector. If this approach is used, it will be imperative to incubate the transformed bacteria and propagate the colonies at or below 25°C to avoid any potential deletions, insertions, or mutations arising from the potential toxic effect of the viral sequences.

The integrity of any DNA used is also crucial for the successful assembly of all five viral fragments into pBeloBAC. During DNA isolation using gel electrophoresis, only brief exposure of the DNA to long-wavelength UV light (e.g., 360 nm) is recommended, since DNA is very vulnerable to UV light, particularly short-wavelength UV light (e.g., 254, 302, or 312 nm). The integrity and degradation of the pBeloBAC plasmid must also be monitored closely, since the pBeloBAC plasmid is prepared from a large volume of bacteria and bacterial enzymes are isolated together with pBeloBAC as well. We recommend that pBeloBAC and its derivates be stored at −80°C instead of classical plasmid storage of −20°C after being isolated and purified.

## MATERIALS AND METHODS

### Equipment.

The equipment needed for this procedures is as follows: −80°C freezer (PHC Corporation, catalog no. MDF-DU900V), 0.2-mL PCR tube (Greiner Bio-One, catalog no. 683201), 0.22-μm syringe filter unit (Millipore, catalog no. SLGP033RS), 1.5-mL tube (Greiner Bio-One, catalog no. 616261), 10-mL serological pipette (Greiner Bio-One, catalog no. 607180), 10-μL filtered tip (Thermo Fisher Scientific, catalog no. 94056980), 100-mm petri dish (Fisher Scientific, catalog no. NC9463569), 1,000-μL filtered tip (Thermo Fisher Scientific, catalog no. 94056710), 1,000-mL conical flask (VWR, catalog no. 470332-218), 15-mL tube (Greiner Bio-One, catalog no. 188201), 2-mm electroporation cuvettes (Bio-Rad, catalog no. 1652092), 200-μL filtered tip (Thermo Fisher Scientific, catalog no. 94056380), 25-mL serological pipette (Greiner Bio-One, catalog no. 760180), 250-mL conical flask (VWR, catalog no. 470332-214), 250 mL polycarbonate centrifuge bottles (Thermo Fisher Scientific, catalog no. 3122-0250), 250-mL Wheaton bottle (VWR, catalog no. 16159-856), 50-mL tube (Greiner Bio-One, catalog no. 227201), 500-mL Wheaton bottle (VWR, catalog no. 16159-889), 6-well plate (Greiner Bio-One, catalog no. 657160), bacterial culture tube (VWR, catalog no. 60818-689), bacterial incubator (VWR, catalog no. 97025-630), Bunsen burner (VWR, catalog no. 89038-530), ChemiDoc imaging system (Bio-Rad, catalog no. 12003153), CO_2_ incubator (PHC Corporation, catalog no. MCO-170AICUVL-PA), electroporator (Bio-Rad, catalog no. 1652100), EVOS imaging system (Thermo Fisher Scientific, catalog no. AMF5000), gel electrophoresis system (Thermo Fisher Scientific, catalog no. A3-1), large-capacity refrigerated centrifuge (Thermo Fisher Scientific, catalog no. 12121680), microcentrifuge (Thermo Fisher Scientific, catalog no. 75002440), orbital shaker (Thermo Fisher Scientific, catalog no. SHKE4450), pH meter (Sartorius, catalog no. PYP102S), pipette set (Gilson, catalog no. F167380), power supply (Bio-Rad, catalog no. 1645050), scale (Sartorius, catalog no. ENTRIS2202-1S), NanoDrop One microvolume UV–visible-spectrum (Vis) spectrophotometer (Thermo Fisher Scientific, catalog no. ND-ONE-W), sterile single-use bottle-top filters (Thermo Fisher Scientific, catalog no. 5954520), T225 flask (Thermo Fisher Scientific, catalog no. 159934), T75 flask (Thermo Fisher Scientific, catalog no. 156499), thermal cycler (G-Storm, catalog no. GS00482), ultrapure water system (Millipore, catalog no. ZIQ7000T0), UV 365-nm transilluminator (UVP, catalog no. 95-0459-01), vacuum manifold (Promega, catalog no. A7231), and water bath (VWR, catalog no. 76308-900).

### Cells.

The cells needed for the procedure are as follows: DH10B electrocompetent cells (Thermo Fisher Scientific, catalog no. 18290015), NEB stable competent cells (New England Biolabs, catalog no. C3040H), and Vero E6 cells (lab-maintained derivative from the American Type Culture Collection [ATCC], catalog no. CRL-1586). It is critical to ensure that all the designated materials used for maintaining Vero E6 cells are not used for other cell lines.

### Reagents.

Reagents needed for the procedure are as follows: 0.05% trypsin–0.53 mM EDTA (Corning, catalog no. 25-051-CI), 0.4% trypan blue (Corning, catalog no. 25-900-CI), 10% formalin solution (Sigma-Aldrich, catalog no. HT501128-4L), 100× penicillin–streptomycin–l-glutamine (PSG) (Corning, catalog no. 30-009-CI), 10,000× GelRed (Biotium, catalog no. 41003-1), 1 Kb Plus DNA ladder (Thermo Fisher Scientific, catalog no. 10787026), 35% bovine serum albumin (BSA) solution (Sigma-Aldrich, catalog no. A7409), 50× Tris-acetate-EDTA (TAE) buffer (Fisher Scientific, catalog no. BP1332500), 6× gel loading dye (New England Biolabs, catalog no. B7024S), ABC-HRP mouse IgG kit (Vector Laboratories, catalog no. PK-4002), agar (Oxoid, catalog no. LP0028), agarose (Lonza, catalog no. 50004), ampicillin (Corning, catalog no. 61-238-RM), ATP-dependent DNase kit (Lucigen, catalog no. E3110K), BamHI-HF (New England Biolabs, catalog no. R3136L), BsaI (New England Biolabs, catalog no. R3733L), BsmBI (New England Biolabs, catalog no. R0739L), BstBI (New England Biolabs, catalog no. R0519L), Cell culture water (Thermo Fisher Scientific, catalog no. A1287301), chloramphenicol (Corning, catalog no. 61-239-RI), chloroform (Fisher Scientific, catalog no. AAJ67241AP), 3,3′-diaminobenzidine (DAB) substrate kit (Vector Laboratories, catalog no. SK-4100), DAPI (diamidino-2-phenylindole) solution (1 mg/mL) (Thermo Fisher Scientific, catalog no. 62248), DEAE-dextran (MP Biomedicals, catalog no. 195133), Dulbecco's modified Eagle medium/nutrient mixture F-12 (DMEM/F-12) powder (Thermo Fisher Scientific, catalog no. 12400024), DMEM (Corning, catalog no. 15-013-CM), ethanol (Sigma-Aldrich, catalog no. 459836-2L), Expand high-fidelity PCR system (Sigma-Aldrich, catalog no. 11759078001), fetal bovine serum (FBS) (VWR, catalog no. 97068-085), first-strand synthesis system for RT-PCR (Thermo Fisher Scientific, catalog no. 11904018), glacial acetic acid (Fisher Scientific, catalog no. 18-600-204), HindIII (New England Biolabs, catalog no. R0104L), isopropanol (Fisher Scientific, catalog no. BP26181), KasI (New England Biolabs, catalog no. R0544L), Luria-Bertani (LB) agar (BD, catalog no. 214010), Lipofectamine 2000 reagent (Thermo Fisher Scientific, catalog no. 11668019), MluI-HF (New England Biolabs, catalog no. R3198L), mouse anti-N protein monoclonal antibody (kindly provided by Thomas Moran from Icahn School of Medicine at Mount Sinai or commercially available from Sigma-Aldrich, catalog no. ZMS1075), NEB buffer set (New England Biolabs, catalog no. B7200S), nuclease-free water (Promega, catalog no. P1193), Opti-MEM (Thermo Fisher Scientific, catalog no. 31985-070), PacI (New England Biolabs, catalog no. R0547L), PciI (New England Biolabs, catalog no. R0655L), PCR cleanup system (Promega, catalog no. A9285), plasmid maxiprep kit (Omega, catalog no. D6922-04), plasmid miniprep kit (Omega, catalog no. D6942-02), phosphate-buffered saline (PBS) (Corning, catalog no. 21-040-CM), QIAEX II gel extraction kit (Qiagen, catalog no. 20021), RsrII (New England Biolabs, catalog no. R0501L), recombinant shrimp alkaline phosphatase (rSAP) (New England Biolabs, catalog no. M0371L), SOC (super optimal broth with catabolite repression) medium (Thermo Fisher Scientific, catalog no. 15544304), sodium acetate (Fisher Scientific, catalog no. BP333-500), sodium bicarbonate (Fisher Scientific, catalog no. S233-500), sodium chloride (Fisher Scientific, catalog no. S271-1), T4 DNA ligase and buffer (New England Biolabs, catalog no. M0202L), TRITC (tetramethyl rhodamine isothiocyanate)-labeled donkey anti-mouse IgG secondary antibody (Thermo Fisher Scientific, catalog no. A16016), Triton X-100 (Sigma-Aldrich, catalog no. X100-500ML), TRIzol (Thermo Fisher Scientific, catalog no. 15596018), tryptone (Thermo Fisher Scientific, catalog no. 211699), and yeast extract (BD, catalog no. 212720).

### Reagent preparation.

For 0.5% Triton X-100 solution, add 50 mL Triton X-100 to 950 mL PBS, mix thoroughly by stirring at room temperature for 1 h, and store at 4°C. For 1% DEAE-dextran, add 1 g of DEAE-dextran powder to 100 mL cell culture water and sterilize by autoclaving at 121°C for 30 min. For 1× TAE buffer, mix 100 mL of 50× TAE solution with 4,900 mL ultrapure deionized water in a sanitized carboy and store at room temperature. For 2× DMEM–F-12 solution, dissolve 1 bag of DMEM–F-12 powder and 1.2 g sodium bicarbonate in 500 mL cell culture water, mix with 10 mL of PSG–6 mL of 35% BSA solution, and sterilize by passing through a sterile single-use bottle-top filter. Keep sterile and store at 4°C. For 2% agar, add 2 g of Oxoid agar to 100 mL cell culture water in a 250-mL Wheaton glass bottle, autoclave at 121°C for 30 min and store at room temperature. The 2% agar will solidify after cooling. Microwave for 1 to 3 min to completely melt before using in the plaque assays. For 75% ethanol, mix 150 mL of absolute ethanol and 50 mL of nuclease-free water in a 250-mL Wheaton glass bottle and store at room temperature. For ampicillin stock (150 mg/mL), add 1.5 g ampicillin to 10 mL of deionized water and mix thoroughly until the powder is fully dissolved in a 50-mL tube, sterilize the solution by passing through a 0.22-μm syringe filter unit, dispense into 0.5-mL aliquots in 1.5-mL tubes, and store the aliquots at −20°C. For blocking solution, add 77 mL of 35% BSA solution to 1 L of PBS, mix completely, and store at 4°C. The blocking solution is also used to dilute primary and secondary antibodies. For chloramphenicol stock (12.5 mg/mL), add 0.25 g chloramphenicol to 20 mL absolute ethanol in a 50-mL tube, vortex until the powder is completely dissolved, aliquot the solution into 1.5-mL tubes containing 0.5 mL per tube and store at −20°C. For complete cell culture medium, add 110 mL FBS and 11 mL 100× PSG to 1 L DMEM and mix completely in a biosafety cabinet, keep the complete cell culture medium at 4°C, and use within 1 month. For LB agar plates containing ampicillin or chloramphenicol, add 4 g sodium chloride, 2 g yeast extract, 4 g tryptone and 6 g LB agar to 400 mL deionized water in a 500-mL Wheaton glass bottle and autoclave at 121°C for 30 min, add 0.4 mL ampicillin stock (final working concentration, 150 mg/mL) or chloramphenicol stock (final working concentration, 12.5 mg/mL) to 400 mL LB agar solution after equilibrating the solution in a 55°C water bath for 30 min, and pour ~25 mL LB agar solution into each 100-mm petri dish. The plates should be wrapped using plastic film, stored at 4°C, and used within 1 month. For LB broth containing ampicillin or chloramphenicol, dissolve 4 g sodium chloride, 2 g yeast extract, and 4 g of tryptone into 400 mL deionized water in a 500-mL Wheaton glass bottle and autoclave at 121°C for 30 min. After cooling, add 0.4 mL ampicillin stock or chloramphenicol stock to 400 mL LB medium in a biosafety cabinet, store the LB medium containing antibiotics at 4°C, and use the prepared LB broth containing ampicillin or chloramphenicol within 1 month. For sodium acetate solution (3.0 M, pH 5.2), dissolve 12.3 g sodium acetate into 30 mL nuclease-free water in a 250-mL Wheaton glass bottle, adjust the pH to 5.2 using glacial acetic acid, adjust the final volume to 50 mL using nuclease-free water, and store at room temperature. For postinfection medium, add 22 mL FBS and 11 mL 100× PSG to 1 L DMEM in a biosafety cabinet and mix completely; keep the postinfection medium at 4°C and use it within 1 month.

### Experimental design.

To clone the SARS-CoV-2 genome into the empty pBeloBAC, we analyzed the entire viral genome sequence of the SARS-CoV-2/human/USA/WA-CDC-02982586-001/2020, WA-1 (GenBank accession no. MN985325) for unique restriction sites using the online NEBcutter program (http://nc2.neb.com/NEBcutter2/) and designed 5 viral fragments (F1 to F5) ~5,000 to 8,000 bp in length (Fig. 1a). Some restriction sites that have dual cutters might need to be manipulated for cloning purposes by replacing them with silent mutations that can be used as genetic tags to distinguish between rSARS-CoV-2 and the natural SARS-CoV-2 isolate. In our case, we mutated a MluI restriction site in the membrane (M) gene in the synthesized F1 and a BstBI restriction site in the spike (S) gene in the synthesized F5 to facilitate the cloning work (see Text S1 in the supplemental material).

It is also imperative to consider that the restriction sites used be unique in the empty pBeloBAC. If a limited number of unique restriction sites are available for efficient and convenient cloning, one potential strategy is to incorporate type IIS restriction sites. The type IIS restriction enzymes recognize asymmetric DNA sequences and could generate compatible overhangs with unique restriction sites. We incorporated this strategy in our protocol: two BsmBI restriction sites were included in the 5′ and 3′ ends of the F1 to generate overhangs compatible with PciI and HindIII sites (Text S1).

After the design of the five fragments is completed, they can be chemically synthesized using any commercial synthesis service and cloned in an appropriate shuttle vector (e.g., pUC57) (Fig. 1b) before being assembled into the empty pBeloBAC (Fig. 1c). Then, the fragments in the shuttle vectors were released and recovered (Fig. 2a), and F1 was first ligated into the linearized pBeloBAC. The pBeloBAC-F1-positive colonies were selected by colony PCR (Fig. 2b) and confirmed by Sanger sequencing. Afterward, F2 to F5 were sequentially cloned into pBeloBAC-F1 using KasI and PacI (F2), PacI and MluI (F3), MluI and BstBI (F4), and BstBI and BamHI (F5). The presence of F2 to F5 was confirmed with restriction digestions (Fig. 2c to f). After confirming that pBeloBAC contained all five fragments (pBeloBAC-FL) by restriction digestion, the pBeloBAC DNA plasmid was prepared in a maxiprep scale for transfection, using Lipofectamine 2000, of Vero E6 cells (Fig. 3a). The ability of using the pBeloBAC-FL plasmid to generate rSARS-CoV-2 was confirmed by assessing cytopathic effect (CPE) in transfected Vero E6 cells (Fig. 3b), by IFA (Fig. 3c), and by Sanger sequencing of RT-PCR products (Fig. 3d).

Next, the Venus reporter gene was introduced into the pUC57-F1 (Fig. 4a) before being subcloned into pBeloBAC-FL (Fig. 4b). To that end, the Venus-2A and the entire pUC57-F1 were amplified by PCR and inverse PCR (Fig. 5a), respectively, and then they were ligated using compatible overhangs generated by BsaI restriction digestions to achieve a scarless pUC57-F1/Venus-2A without introducing any extra sequences. To generate pBeloBAC-FL/Venus-2A, F1 containing Venus-2A was released and subcloned into pBeloBAC-FL using the restriction sites of BamHI and RsrII (Fig. 5b). Finally, positive pBeloBAC-FL/Venus-2A clones were selected by colony PCR (Fig. 5c), and maxiprep plasmids were prepared and transfected into Vero E6 cells using Lipofectamine 2000 to generate rSARS-CoV-2/Venus-2A. The expression of Venus in Vero E6 cells infected with the supernatant of the transfected Vero E6 cells confirmed the rescue of rSARS-CoV-2/Venus-2A (Fig. 6a). The successful detection of fluorescent plaques with a ChemiDoc imaging system further confirmed that the rescued rSARS-CoV-2/Venus-2A expressing a robust level of Venus fluorescent expression in all the viral plaques by comparing to those detected by immunostaining using an antibody against the viral N protein (Fig. 6b).

### Experimental procedures. Section 1: assembly of the full-length SARS-CoV-2 genome sequences into empty pBeloBAC to generate pBeloBAC-FL (time, 18 days).

**(i) Preparation of the five fragments (time, 1 day).**

Step 1: set up a 100-μL digestion reaction in a 0.2-mL PCR tube with appropriate restriction enzymes for each plasmid with the following: nuclease-free water, up to 100 μL; 10× buffer, 10 μL; plasmids, 5 μg, and restriction enzymes, 20 U each. Note that F1 is released by a single-restriction digestion with BsmBI and generates overhangs compatible with those of PciI and HindIII.

Step 2: incubate the mixture at 37°C in a thermal cycler for 3 h. Be aware that BsmBI and BstBI work at 55°C and 65°C, respectively.

(i) Prepare five 0.6% agarose gels that are 10 cm long and with 10-mm-wide loading wells.

(ii) Weigh 0.6 g agarose powder and dispense into a conical 250-mL glass flask. Add 100 mL 1× TAE buffer.

(iii) After gently mixing, microwave for 3 min to completely melt the agarose.

(iv) Add 10 μL of 10,000× GelRed to the 0.6% agarose solution after the gel’s temperature has cooled down to 50°C. Mix thoroughly.

(v) Pour the mixture into a gel casting tray which has been fitted with a gel comb. The agarose gel should be used after solidifying.

(vi) Place the solidified agarose gel into an electrophoresis tank and add enough 1× TAE buffer to ensure that the gel is fully submerged.

Step 3: stop restriction digestion reaction by adding 20 μL of 6× gel loading dye to each tube and mix thoroughly.

Step 4: load each DNA sample to 3 wells (40 μL/well) using a P100 pipette and load 10 μL of 1 Kb Plus DNA ladder in a separate well.

Step 5: separate the DNA fragments with electrophoresis at 60 V until bands are adequately separated.

Step 6: recover the DNA fragments from the gel using a QIAEX II gel extraction kit. QIAEX II particles provide a slurry for gel extraction and ensure efficient recovery without shearing, even for large DNA fragments.

(i) Excise the target DNA bands in the gel by visualizing with a UV transilluminator and place each gel slice into a 2-mL tube. Always wear a UV protection mask when the UV transilluminator is on.

(ii) Weigh the gel slice; add 3 volumes of buffer QX1 and 2 volumes of nuclease-free water. Note that the weight of a 100-mg gel slice corresponds to a volume of 100 μL.

(iii) Add 30 μL of vortexed QIAEX II to each tube.

(iv) Incubate the tubes at 50°C for 10 min to solubilize the agarose gel slices and to bind the DNA. Mix by flicking and inverting the tube 5 times every 2 min until completely solubilized.

(v) Centrifuge the samples for 30 s and carefully remove supernatant with a P1000 pipette.

(vi) Add 500 μL buffer QX1; resuspend the pellet by flicking and inverting the tube 5 times.

(vii) Centrifuge the sample for 30 s and remove the supernatant with a P1000 pipette.

(viii) Add 500 μL buffer PE; resuspend the pellet by flicking and inverting the tube 5 times.

(ix) Centrifuge the sample for 30 s and remove the supernatant with a P1000 pipette.

(x) Add 500 μL buffer PE; resuspend the pellet by flicking and inverting the tube 5 times.

(xi) Centrifuge the sample for 30 s, remove the supernatant with a P1000 pipette, and carefully remove all traces of supernatant with a P10 pipette.

(xii) Air dry the sample until a white pellet is visualized (~20 min). Do not overdry, as this may lead to decreased elution efficiency.

(xiii) Add 30 μL nuclease-free water to resuspend the pellet by flicking 5 times. Incubate the tube at 50°C for 10 min.

(xiv) Centrifuge for 30 s. Carefully pipette the supernatant into a clean 1.5-mL tube.

Step 7: measure the concentration using a NanoDrop instrument, and run 1 μL of each fragment in a 0.6% agarose gel to check the size of DNA fragments. Image the gel using a ChemiDoc imaging system. The expected bands for F1 to F5 are shown in Fig. 2a.

**(ii) Preparation of empty pBeloBAC (time, 2 days).**

Step 8: electroporate the empty pBeloBAC plasmid into DH10B electrocompetent cells.

(i) Thaw a vial of DH10B electrocompetent cells on ice, dispense 40 μL to the tube containing 10 ng of the empty pBeloBAC DNA, and add deionized water to a final volume of 100 μL.

(ii) Transfer the 100 μL DH10B-DNA mixture into a prechilled 2-mm electroporation cuvette.

(iii) Pulse the DH10B-DNA mixture once with a MicroPulser electroporator using the EC2 program (2.5 kV, 10 μF, 600 Ω).

(iv) Immediately add 900 μL SOC medium to the electroporation cuvette, pipette up and down gently 3 times, and move all the cells into a bacterial culture tube.

(v) Incubate the bacterial culture tube at 37°C with shaking at 250 rpm in an orbital shaker for 1 h.

(vi) Spread all bacteria (200 μL/plate) on prewarmed LB plates containing chloramphenicol.

(vii) Incubate the plates invertedly at 37°C for 16 h.

Step 9: pick a single colony and place in 200 mL LB broth that contains chloramphenicol in a 1,000-mL conical flask. Keep the flask at 37°C with 250 rpm shaking in an orbital shaker for 16 h.

Step 10: carry out maxiprep of empty pBeloBAC.

(i) Pellet bacteria in a 250-mL bottle by centrifuging at 12,000 × *g* for 5 min at 4°C.

(ii) Resuspend cells in 48 mL solution I-RNase A.

(iii) Add 48 mL solution II and invert gently to obtain a clear lysate.

(iv) Add 64 mL ice-cold N3 buffer and mix gently by inverting the tube 10 times until a flocculent white precipitate forms. Incubate on ice for 10 min.

(v) Spin down the precipitate by centrifuging at 15,000 × *g* for 15 min at 4°C.

(vi) Clarify the supernatant by passing through a syringe filter.

(vii) Pass the clarified supernatant through a maxiprep column which is inserted in a vacuum manifold. The maximum vacuum (negative) pressure is 150 mm Hg.

(viii) Add 10 mL HBC buffer to the column, switch on the vacuum source to draw the solution through the column, and then switch off the vacuum source.

(ix) Add 10 mL wash buffer to the column, switch on the vacuum source to draw the solution through the column, and then switch off the vacuum source.

(x) Add another 10 mL wash buffer to the column, switch on the vacuum source to draw the solution through the column, and then switch off the vacuum source.

(xi) Put the column back into a 50-mL collection tube and centrifuge at 12,000 × *g* for 5 min at room temperature.

(xii) Elute DNA into a new 50-mL tube by adding 1 mL nuclease-free water to the column and centrifuging at 12,000 × *g* for 5 min at room temperature.

(xiii) Using a NanoDrop instrument, determine the concentration, which is usually ~25 ng/μL.

Step 11: purify the empty pBeloBAC using an ATP-dependent DNase kit to remove the contaminated bacterial genome fragments. The pBeloBAC plasmids that are used for cloning must be purified.

(i) Set up a purification reaction in a 2.0-mL tube as follows: carry out maxiprep of plasmid in nuclease-free water (420 μL), 10× reaction buffer (50 μL), ATP (25 mM; 20 μL), and plasmid-safe DNase (10 μL).

(ii) Incubate the tube at 37°C for 3 h.

(iii) Inactivate plasmid-safe DNase by incubating at 70°C for 30 min.

(iv) Add 50 μL of sodium acetate (3 M, pH 5.2) and 1.25 mL of absolute ethanol to the reaction mixture.

(v) After mixing thoroughly, freeze the solution at −80°C for 30 min.

(vi) Centrifuge at 15,000 × *g* for 10 min at 4°C.

(vii) Decant supernatant, and resuspend the pellet in 1 mL of 75% ethanol.

(viii) Centrifuge at 15,000 × *g* for 5 min at 4°C.

(ix) Discard supernatant and remove trace ethanol with a P100 pipette.

(x) Air dry the pellet for 5 min, dissolve the pellet into the desired volume of nuclease-free water, and measure concentration using a NanoDrop instrument.

Step 12: aliquot the purified empty pBeloBAC and store at −80°C.

**(iii) Preparation of the linearized empty pBeloBAC (time, 1 day).**

Step 13: set up a digestion reaction of the purified empty pBeloBAC in a 0.2-mL tube with the following: nuclease-free water, up to 100 μL; 10× 2.1 buffer, 10 μL; purified empty pBeloBAC, 3 μg; PciI, 15 U; HindIII, 10 U; and rSAP, 10 U. The rSAP is included in the digestion reaction to minimize the self-ligation of the linearized backbone plasmid.

Step 14: incubate the mixture at 37°C for 3 h.

Step 15: prepare a 0.6% agarose gel as described in step 2.

Step 16: stop the digestion reaction by adding 20 μL of 6× gel loading dye to each tube, mixing thoroughly.

Step 17: load the sample (40 μL/well) into a 0.6% agarose gel using a P100 pipette and load 10 μL of 1 Kb Plus DNA ladder in a separate well.

Step 18: separate the DNA fragments by electrophoresis at 60 V until bands are adequately separated.

Step 19: recover the linearized pBeloBAC from the gel as described in step 6. The DNA recovery rate is ~50%.

Step 20: after determining the concentration, aliquot the linearized empty pBeloBAC and store at −80°C.

**(iv) Assembly of the F1 into the linearized pBeloBAC (time, 2 days).**

Step 21: set up a ligation reaction in a 0.2-mL tube for assembling the F1 into the linearized empty pBeloBAC with the following: nuclease-free water, up to 100 μL; 10× T4 ligation buffer, 10 μL; F1, 200 ng; linearized empty pBeloBAC, 100 ng; and T4 ligase, 10 μL.

Step 22: incubate the ligation mixture at room temperature for 3 h.

Step 23: precipitate DNA from the ligation reaction mixture.

(i) Add 10 μL of sodium acetate solution (3.0 M, pH 5.2) and 250 μL of absolute ethanol to the tube, and incubate at −80°C for 30 min.

(ii) Centrifuge at 15,000 × *g* for 10 min at 4°C.

(iii) Discard the supernatant, and resuspend the pellet with 1 mL of 75% ethanol.

(iv) Centrifuge at 15,000 × *g* for 5 min at 4°C.

(v) Decant the supernatant, remove the trace fluid by using a P100 pipette, and air dry for 5 min.

(vi) Dissolve the pellet in 60 μL nuclease-free water and immerse the tube in ice.

Step 24: electroporate the ligated DNA into DH10B electrocompetent cells. 

(i) Thaw a vial of DH10B electrocompetent cells; dispense 40 μL to the tube containing 60 μL of dissolved ligated DNA.

(ii) Transfer the 100-μL DH10B-DNA mixture into a prechilled 2-mm electroporation cuvette.

(iii) Pulse the DH10B-DNA mixture once with a MicroPulser electroporator using the EC2 program.

(iv) Immediately add 900 μL SOC medium to the electroporation cuvette, pipette up and down gently 3 times, and move all the cells into a bacterial culture tube.

(v) Incubate the bacterial culture tube at 37°C with shaking at 250 rpm in an orbital shaker for 1 h.

(vi) Spread all bacteria on four prewarmed LB plates containing chloramphenicol (250 μL/plate).

(vii) Incubate the plates invertedly at 37°C for 16 h. 

Step 25: pick 6 colonies and place each colony into a separate culture tube with 1 mL LB medium containing chloramphenicol.

Step 26: incubate the tubes at 37°C with shaking at 250 rpm in an orbital shaker for 3 h.

Step 27: prepare a colony PCR master mix in a 1.5 mL tube according to the recipe as follows: nuclease-free water, 33 μL; 10× buffer without MgCl_2_, 5 μL; MgCl2 (25 mM), 3 μL; deoxynucleoside triphosphate (dNTP) (10 mM mix), 1 μL; F1-F (5 μM), 3 μL; F1-R (5 μM), 3 μL; and Expand high fidelity enzyme, 1 μL. The sequences of the primers used in this colony PCR are listed in Table 1. 

Step 28: mix thoroughly, dispense 49 μL of the PCR into 0.2-ml tubes, and add 1 μL of each cultured bacterium into tubes. Include a tube with 1 μL of nuclease-free water as a negative control. Also, include a tube with 1 μL with 10 ng of pUC57-F1 plasmid as a positive control.

Step 29: place the tubes in a thermal cycler and start the PCR using the following conditions: initial denaturation (94°C), 2 min, 1 cycle; denaturation (94°C), 30 s, 30 cycles; annealing (50°C), 30 s, 30 cycles; elongation (72°C), 3 min, 30 cycles; final elongation (72°C), 10 min, 1 cycle; and cooling (4°C), unlimited time.

Step 30: confirm PCR amplification in a 0.6% agarose gel. An example result for colony PCR for cloning of F1 into pBeloBAC is shown in Fig. 2b. The PCR-positive clone is further confirmed by Sanger sequencing using the CMV/F and bGH/R primers (Table 1) and designated pBeloBAC-F1.

**TABLE 1:** Primers

Purpose	Primer	Sequence (5′→3′)
Colony PCR screening	F1-F	ATGTCTGATAATGGACCCCAAAATCAGCGAAAT
	F1-R	TTAGGCCTGAGTTGAGTCAGCACTGCTC
	F2-F	GGGAGAAACACTTCCCACAGAAGTGTTAACAG
	F2-R	GCTGATAATAATGGTGCAAGTAGAACTTCGTGCTG
	F3-F	GGCTTGATGACGTAGTTTACTGTCCAAGACATG
	F3-R	TCCTAGCACCATCATCATACACAGTTCTTGCT
	F4-F	GGATGGTAATGCTGCTATCAGCGATTATGACT
	F4-R	GGATTCTTGATGGATCTGGGTAAGGAAGGTACA
	F5-F	GGCTGCGTTATAGCTTGGAATTCTAACAATCTTGAT
	F5-R	AGTAGATCTTCAATAAATGACCTCTTGCTTGGTTTTG
	Venus-F	ATGGTGAGCAAGGGCGAGGAGCTGTT
	Venus-R	TTTGTACAGCTCGTCCATGCCGAGAGT

Sanger sequencing	CMV/F	CGCAAATGGGCGGTAGGCGTG
	bGH/R	TAGAAGGCACAGTCGAGG
	F1/F	AAGGAGCTGGTGGCCATAGTTAC
	ORF3a/F	ATGGATTTGTTTATGAGAATCTTCACAATTGGAACT
	E/F	ATGTACTCATTCGTTTCGGAAGAGACAG
	M/F	ATGGCAGATTCCAACGGTACTATTACC
	ORF6/F	ATGTTTCATCTCGTTGACTTTCAGGTTACTAT
	ORF8/F	ATGAAATTTCTTGTTTTCTTAGGAATCATCACAAC
	Venus/N	CGTCGCCGTCCAGCTCGACCAG
	Venus/C	CATGGTCCTGCTGGAGTTCGTG
	N/F	ATGTCTGATAATGGACCCCAAAATCAGCGAAAT
	N/R	TTAGGCCTGAGTTGAGTCAGCACTG
	M13/R	GGAAACAGCTATGACCATG

RT-PCR	M-F	ATGGCAGATTCCAACGGTACTATTACCGTT
	M-R	TTACTGTACAAGCAAAGCAATATTGTCACTGCTACT

Generation of rSARS-CoV-2/Venus-2A	Venus-2A-F	GAGGGTCTCTTAAAATGGTGAGCAAGGGCGAGGAGCTGTTCAC
	Venus-2A-R	ATAGGTCTCTCAGAAGGACCGGGGTTTTCTTCCACGTCCCCTGCTTGCTTTAACAGAGAGAAGTTCGTGGCTCCGGACCCTTTGTACAGCTCGTCCATGCCGAG
	pUC57F1-F	ATAGGTCTCTTCTGATAATGGACCCCAAAATCAGCGAAATGC
	pUC57F1-R	CGCGGTCTCTTTTAGTTTGTTCGTTTAGATGAAATCTAAAACAACACGAACGT

**(v) Sequential assembly of F2 to F5 into pBeloBAC-F1 (time, 12 days).**

Step 31: carry out maxiprep and purify pBeloBAC-F1 similar as described in steps 10 and 11.

Step 32: set up a restriction digestion of PacI and MluI to linearize the purified pBeloBAC-F1, similar to step 13. Recover the linearized pBeloBAC-F1 from a 0.6% gel as described in step 6.

Step 33: assemble F3 into the linearized pBeloBAC-F1, similar to steps 21 to 26. Colony PCR screen for positive clones using the primers of F3-F and F3-R (Table 1).

Step 34: confirm insertion of F3 in PCR positive clones by digesting with PacI and MluI; then name the positive clone pBeloBAC-F13, which contains F1 and F3. An example of the expected pBeloBAC-F13 digestion with PacI and MluI is shown in Fig. 2c.

Step 35: next, assemble F4 (using MluI and BstBI), F2 (using KasI and PacI), and F5 (using BstBI and BamHI) into pBeloBAC-F13 one by one as previously described for F3 into pBeloBAC-F1. Confirm by using both colony PCR and restriction digestion. The number of bacterial colonies may range from 100 to 1,000. However, the number of bacterial colonies decrease with the addition of F2 to F5. Expected restriction analysis results of the sequential cloning of F4, F2, and F5 are shown in Fig. 2d to f. The resultant plasmids are named, respectively, pBeloBAC-F134, pBeloBAC-F1342, and pBeloBAC-FL, which contains F1, F2, F3, F4, and F5. Notice that the insertion order of fragments into pBeloBAC-F1 is flexible, and we assemble them based on the order of the plasmids we receive from the company we used for chemical synthesis.

### Section 2: rescue of rSARS-CoV-2 using pBeloBAC-FL (time, 8 days).

**(i) Transfection of pBeloBAC-FL into Vero E6 cells (time, 4 days).**

Step 36: scale up the DH10B harboring pBeloBAC-FL to 200 ml of LB medium containing chloramphenicol in a 1-L flask by inoculating 200 μl from 3 positive-colony PCR clones. Culture at 37°C with 250 rpm in an orbital shaker for 16 h.

Step 37: carry out maxiprep of pBeloBAC-FL as described in step 10. The concentration of pBeloBAC-FL obtained from a maxiprep is usually ~50 ng/μL.

Step 38: transfect pBeloBAC-FL into Vero E6 cells in a 6-well plate. Transfection to generate rSARS-CoV-2 using pBeloBAC-FL must be performed in a biosafety level 3 (BSL-3) laboratory following appropriated institutional biosafety guidelines.

(i) Dilute 10 μL of Lipofectamine 2000 in 250 μL Opti-MEM in a 1.5-mL tube, and incubate at room temperature for 5 min.

(ii) Dilute 4 μg of pBeloBAC-FL in 250 μL Opti-MEM in a 1.5-mL tube. We recommend transfecting 3 different clones of the plasmid to ensure a successful virus rescue.

(iii) Add the diluted Lipofectamine 2000 to the diluted pBeloBAC-FL.

(iv) Incubate the pBeloBAC-FL–Lipofectamine 2000 mixture at room temperature for20 to 30 min.

(v) During the incubation, prepare a Vero E6 cell suspension and seed cells in a 6-well plate (~5 × 10^5^ cells/well) in complete cell culture medium after counting with Trypan blue staining. Add the pBeloBAC-FL–Lipofectamine 2000 mixture into the wells and gently shake back and forth 5 times.

(vi) Incubate the cell plate at 37°C in a CO_2_ incubator.

Step 39: replace the medium with postinfection medium (4 mL/well) at 24 h posttransfection.

Step 40: at 72 h posttransfection, collect the supernatant of transfected Vero E6 cells and dispense 1 mL/well to infect 2 different wells of fresh Vero E6 cell monolayers in a 6-well plate for confirmation of virus rescue using IFA and RT-PCR.

**(ii) Confirmation of rSARS-CoV-2/WT rescue (time, 4 days).**

Step 41: at 48 h postinfection (hpi), check the infected cells using an EVOS imaging system for CPE. An example of expected CPE is shown in Fig. 3b.

Step 42: discard the supernatant. Add 1 mL of TRIzol to one well of the infected cells for RNA extraction, and fix the other well of infected cells with 10% formalin solution for IFA. The TRIzol-treated sample and the 10% formalin-fixed plate could now be moved to BSL-2 by complying with proper biosafety guidelines and institutional policy.

Step 43: analyze the 10% formalin-fixed cells by IFA.

(i) Permeabilize the cells using 0.5% Triton X-100 (2 mL/well) for 10 min at room temperature.

(ii) Block the cells using blocking solution (1 mL/well) for 30 min at 37°C.

(iii) Dilute the primary antibody against viral N protein (1C7C7) in blocking solution at a final concentration of 1 μg/mL, and incubate with the cells (1 mL/well) at 37°C for 1 h.

(iv) Wash the cells 3 times with PBS.

(v) Dilute the TRITC-labeled donkey anti-mouse IgG secondary antibody in blocking solution to a final concentration of 1 μg/mL, and incubate with the cells (1 mL/well) at 37°C for 1 h.

(vi) Wash the cells 3 times with PBS.

(vii) Dilute the DAPI solution in PBS to a final concentration of 1 μg/mL, and incubate with the cells (1 mL/well) at 37°C for 10 min.

(viii) Wash the cells 3 times with PBS.

(ix) Observe and photograph the cells using an EVOS imaging system. An example of an IFA result is shown in Fig. 3c.

Step 44: during the immunostaining, extract RNA from the TRIzol-treated cells.

(i) Add 200 μL of chloroform to 1 mL of TRIzol-treated sample and mix thoroughly by vigorous shaking. Do not vortex.

(ii) Centrifuge the sample for 15 min at 15,000 × *g* at 4°C. The mixture should separate into a lower red phenol-chloroform phase, an interphase, and a colorless upper aqueous phase.

(iii) Collect the aqueous phase containing the RNA into a new 1.5-mL tube.

(iv) Add 500 μL of isopropanol, mix thoroughly by inverting the tube 10 times, and incubate for 5 min at room temperature.

(v) Centrifuge for 10 min at 15,000 × *g* at 4°C.

(vi) The total RNA precipitate should form a white gel-like pellet at the bottom of the tube.

(vii) Decant the supernatant, and resuspend the pellet in 1 mL of 75% ethanol.

(viii) Centrifuge at 15,000 × *g* for 5 min at 4°C.

(ix) Decant the supernatant, and remove trace ethanol using a P100 pipette.

(x) Air dry the RNA pellet for 5 min at room temperature. Do not overdry the RNA, since it will be difficult to dissolve.

(xi) Resuspend the pellet in 100 μL nuclease-free water and determine the concentration using a NanoDrop instrument. Proceed to downstream applications or store the RNA at −80°C for less than 1 month.

Step 45: set up an RT reaction to convert RNA to cDNA.

(i) Combine the following components in a 0.2-mL tube: RNA, 2 μg; random primers (2 μM), 1 μL; dNTP (10 mM mix), 1 μL; and nuclease-free water, up to 10 μL.

(ii) Incubate the mixture at 65°C for 5 min; then immediately place it on ice for 1 min.

(iii) Add 10× RT buffer (2 μL) MgCl_2_ (25 mM; 4 μL), dithiothreitol (DTT; 100 mM; 2 μL), and RNase inhibitor (40 U/μL; 1 μL) to each RNA/primer mixture, mix gently, and collect by brief centrifugation.

(iv) Incubate at 25°C for 2 min.

(v) Add 1 μl of the SuperScript II reverse transcriptase to each tube and incubate at 42°C for 50 min.

(vi) Terminate the reaction by incubating at 70°C for 15 min. Proceed to downstream applications or store the cDNA at −20°C.

Step 46: set up a PCR in a 0.2-mL tube with the following: nuclease-free water, 33 μL; 10× buffer without MgCl_2_, 3 μL; dNTP (10 mM mix), 1 μL; M-F (5 μM), 3 μL; M-R (5 μM), 3 μL; cDNA, 1 μL; and Expand high fidelity enzyme, 1 μL. The sequences of the primers are listed in Table 1.

Step 47: place the tube in a thermal cycler and start the cycling using the following conditions: initial denaturation (94°C), 2 min, 1 cycle; denaturation (94°C), 30 s, 30 cycles; annealing (55°C), 30 s, 30 cycles; elongation (72°C), 6 min, 30 cycles; final elongation (72°C), 6 min, 30 cycles; and cooling (4°C), unlimited time.

Step 48: confirm PCR amplification in a 0.6% agarose gel and recover the target DNA band from the gel as described in step 6.

Step 49: sequence the PCR product using the M/F primer (Table 1). The expected results of sequencing the M gene from rSARS-CoV-2/WT are shown in Fig. 3d.

### Section 3: cloning of Venus reporter gene into pBeloBAC-FL (time, 6 days).

**(i) Cloning of Venus into pUC57-F1 (time, 3 days).**

Step 50: set up a PCR to amplify Venus-2A in a 0.2-mL tube with the following: nuclease-free water, 33 μL; 10× buffer without MgCl_2_, 5 μL; MgCl_2_ (25 mM), 3 μL; dNTP (10 mM mix), 1 μL; Venus-2A-F (5 μM), 3 μL; Venus-2A-R (5 μM), 3 μL; Venus template, 1 μL; and Expand high-fidelity enzyme, 1 μL. The sequences of the primers are listed in Table 1.

Step 51: place the tube in a thermal block cycler and start the cycling using the thermal profile described in step 47.

Step 52: recover the amplified Venus-2A from the gel as described in step 6.

Step 53: set up a PCR to amplify pUC57F1in a 0.2-mL tube with the following: nuclease-free water, 32 μL; 10× buffer without MgCl_2_, 5 μL; MgCl_2_ (25 mM), 3 μL; dNTP (10 mM mix), 2 μL; pUC57F1-F (5 μM), 3 μL; pUC57F1-R (5 μM), 3 μL; pUC57-F1, 1 μL; and Expand high-fidelity enzyme, 1 μL. The sequences of the primers are listed in Table 1.

Step 54: place the tube in a thermal block cycler and start the cycling using the thermal profile described in step 47.

Step 55: recover the amplified pUC57F1 from the gel as described in step 6.

Step 56: run 1 μL of each DNA product in a 0.6% agarose gel to check the size. Image the gel using a ChemiDoc imaging system. The expected bands for the PCR products are shown in Fig. 5a.

Step 57: set up digestion reactions for the purified PCR products of Venus-2A and pUC57F1 with the following: nuclease-free water, up to 100 μL; 10× 3.1 buffer, 10 μL; purified Venus-2A or pUC57F1 DNA, 4 μg; and BsaI, 10 U.

Step 58: incubate the reaction mixture at 37°C for 3 h.

Step 59: recover the DNA using a PCR clean-up kit. Be aware that there are 2 bands after BsaI digestion of the amplified pUC57F1; cut and combine these 2 bands into one tube if using gel purification procedures.

(i) Add 3 volumes membrane binding solution of PCR, mix thoroughly.

(ii) Pass the mixture through a column by centrifuging at 15,000 × *g* for 10 s.

(iii) Wash the column twice with 75% ethanol solution.

(iv) Centrifuge at 15,000 × *g* for 1 min to remove trace ethanol.

(v) Elute DNA by adding 30 μL nuclease-free water.

Step 60: set up a ligation reaction to assemble Venus-2A into pUC57F1 with the following: nuclease-free water, up to 20 μL; 10× T4 ligation buffer, 2 μL; pUC57F1, 50 ng; Venus-2A, 20 ng; and T4 ligase, 1 μL.

Step 61: incubate the ligation reaction at room temperature for 1 h.

Step 62: transform the ligated DNA into NEB stable competent cells.

(i) Thaw 1 tube of NEB stable competent cells on ice.

(ii) Add 10 μL of ligation solution to the competent cells, and carefully flick the tube 3 times to mix cells and DNA.

(iii) Place the mixture on ice for 30 min.

(iv) Heat shock the cells in the mixture at exactly 42°C for exactly 30 s.

(v) Immediately put the tubes back on ice for 5 min.

(vi) Pipette 950 μL of room temperature SOC medium into the mixture and place the mixture at 37°C with shaking at 250 rpm in an orbital shaker for 60 min.

(vii) Spread 100 μL of cells onto prewarmed LB agar plates containing ampicillin.

(viii) Incubate plates invertedly at 37°C for 16 h.

Step 63: pick three colonies and place each colony in 5 mL of LB broth containing ampicillin in a bacterial culture tube and culture at 37°C with shaking at 250 rpm in an orbital shaker for 16 h.

Step 64: carry out miniprep of plasmids using an Omega miniprep kit.

(i) Pellet the bacterial culture in a 2.0-mL tube at 15,000 × *g* at 4°C for 1 min.

(ii) Resuspend cells in 500 μL solution I-RNase A.

(iii) Add 500 μL solution II and invert gently to obtain a clear lysate.

(iv) Add 700 μL ice-cold N3 buffer, mix gently by inverting tube 10 times until a white precipitate forms, and incubate the tube on ice for 10 min.

(v) Spin down the precipitate by centrifuging at 15,000 × *g* at 4°C for 10 min.

(vi) Pass the clarified supernatant through a miniprep column which is inserted in a vacuum manifold. The maximum vacuum (negative) pressure is 150 mm Hg.

(vii) Add 500 μL HBC buffer to the column, switch on the vacuum source to draw the solution through the column, and then switch off the vacuum source.

(viii) Add 700 μL wash buffer to the column, switch on the vacuum source to draw the solution through the column, and then switch off the vacuum source.

(ix) Add another 700 μL wash buffer to the column, switch on the vacuum source to draw the solution through the column, and then switch off the vacuum source.

(x) Put the column back into a collection tube and centrifuge at 15,000 × *g* at room temperature for 5 min.

(xi) Elute DNA into a new 1.5-mL tube by adding 100 μL nuclease-free water to the column and centrifuge at 15,000 × *g* at room temperature for 5 min. Using a NanoDrop instrument, determine the concentrations of the plasmids, which are usually 100 to 200 ng/μL.

Step 65: sequence the entire plasmid using the primers listed in Table 1. The positive plasmid is designated pUC57-F1/Venus-2A.

**(ii) Cloning of Venus into pBeloBAC-FL (time, 3 days).**

Step 66: set up a digestion reaction for pUC57-F1/Venus-2A with the following: nuclease-free water, up to 100 μL; 10× 3.1 buffer, 10 μL; pUC 57-F1/Venus-2A, 5 μg; BsaI, 15 U; BamHI, 15 U; and RsrII, 15 U.

Step 67: incubate the reaction mixture at 37°C for 3 h.

Step 68: separate the digested DNA on a 0.6% agarose by electrophoresis at 60 V until bands are adequately separated (Fig. 5b, left lane) and recover the top DNA (F1/Venus-2A) as described in step 6. The DNA recovery rate is ~50%.

Step 69: elute target DNA in 30 μL nuclease-free water and measure the concentration using a NanoDrop instrument.

Step 70: carry out maxiprep of pBeloBAC-FL as described in step 10.

Step 71: purify pBeloBAC-FL as described in step 11.

Step 72: set up a digestion reaction in a 0.2 mL tube to linearize the purified pBeloBAC-FL with the following: nuclease-free water, up to 100 μL; 10× 3.1 buffer, 10 μL; purified pBeloBAC-FL, 50 μg; BamHI, 30 U; RsrII, 30 U; and rSAP, 30 U.

Step 73: incubate the reaction mixture at 37°C for 3 h.

Step 74: stop the reaction by adding 20 μL of 6× gel loading dye and mix thoroughly by pipetting gently up and down 3 times.

Step 75: load the mixture on a 0.6% agarose gel, and separate the bands by electrophoresis at 60 V until bands are adequately separated (Fig. 5b, right lane). Do not load more than 30 μL per well; otherwise, the digested DNA could get stuck in the well.

Step 76: recover the pBeloBAC-FL backbone band as described in step 6.

Step 77: elute the linearized pBeloBAC-FL in 100 μL nuclease-free water and measure the concentration using a NanoDrop instrument. The DNA recovery rate is ~50%.

Step 78: aliquot the linearized pBeloBAC-FL and store at −80°C.

Step 79: set up a ligation reaction for cloning F1/Venus-2A into the digested pBeloBAC-FL in a 1.5 mL tube with the following: nuclease-free water, up to 100 μL; 10× T4 buffer, 10 μL; pBeloBAC-FL backbone, 200 ng; F1/Venus-2A, 200 ng; and T4 ligase, 10 μL.

Step 80: incubate the reaction mixture at room temperature for 6 h.

Step 81: recover the ligated DNA as described in step 23.

Step 82: electroporate the ligated DNA into DH10B cells as described in step 24.

Step 83: pick 6 colonies and place each in a bacterial culture tube that contains 1 mL LB broth containing chloramphenicol.

Step 84: culture at 37°C for 3 h.

Step 85: colony PCR screen for positive clones as described above using the primers binding to F5 (F5-F and F5-R) and Venus (Venus-F and Venus-R) (Table 1). The positive clone is named pBeloBAC-FL/Venus-2A. An example of PCR screening of positive pBeloBAC-FL/Venus-2A clones is shown in Fig. 5c.

### Section 4: recovery of the Venus-expressing rSARS-CoV-2 (rSARS-CoV-2/Venus-2A) (time, 8 days).

**(i) Rescue of rSARS-CoV-2/Venus-2A (time, 4 days).**

Step 86: inoculate 100 μL of PCR-positive bacteria into 100 mL LB broth containing chloramphenicol in a 1-L flask. Culture at 37°C with shaking at 250 rpm in an orbital shaker for 16 h. We usually select 3 PCR-positive clones to expand.

Step 87: carry out maxiprep of pBeloBAC-FL/Venus-2A as described in step 10.

Step 88: transfect Vero E6 cells with the pBeloBAC-FL/Venus-2A as described in step 38.

Step 89: replace the medium with postinfection medium (4 mL/well) at 24 h posttransfection.

Step 90: return the plates to the 37°C CO_2_ incubator.

Step 91: check the transfected cells under an EVOS imaging system for fluorescent Venus expression and collect the supernatant at 72 h posttransfection.

**(ii) Characterization of rSARS-CoV-2/Venus-2A (time, 4 days).**

Step 92: infect fresh confluent Vero E6 cells in a 6-well plate by adding 1 mL/well of the supernatant of the transfected cells for 72 h.

Step 93: analyze the supernatant of the transfected cells by plaque assay.

(i) Dilute the supernatant in 10-fold serial dilutions using postinfection medium.

(ii) Infect monolayers of Vero E6 cells in a 6-well plate with the diluted supernatant for 1 h at 37°C.

(iii) During viral infection, prepare the semisolid medium as follows: cell culture water, 9.5 mL; 2× DMEM–F-12, 25 mL; 1% DEAE-dextran, 0.5 mL; and 2% agar, 15 mL. It is important to melt the agar completely in a microwave oven and mix thoroughly before adding to the medium.

(iv) Discard the infection supernatant and wash cells 3 times with PBS.

(v) Overlay the cells with 4 mL of semisolid medium. Ensure the temperature of the semisolid medium does not exceed 42°C before overlaying the cells.

(vi) Solidify the medium by leaving the plates at room temperature for 10 min.

(vii) Incubate the plates invertedly at 37°C for 72 h in a 5% CO_2_ incubator.

Step 94: fix the plates in 10% formalin. The 10% formalin-fixed plates could now be moved to BSL-2 by complying with institutional biosafety policy.

Step 95: analyze the freshly infected cells by IFA as described in step 43. The expected results of Vero E6 cells infected with rSARS-CoV-2/Venus-2A stained with the monoclonal antibody (MAb) 1C7C7 against the viral N protein are shown in Fig. 6a.

Step 96: remove the agar from the plate used for plaque assay, and image the cells with a ChemiDoc imager. Expected results of Venus expression in the ChemiDoc from Vero E6 cells infected with rSARS-CoV-2/WT and rSARS-CoV-2/Venus-2A are shown in Fig. 6b (left).

Step 97: visualize the plaques by immunostaining.

(i) Permeabilize cells with 2 mL of 0.5% Triton X-100 at room temperature for 10 min.

(ii) Block the cells by incubating in blocking solution (1 mL/well) at 37°C for 30 min.

(iii) Dilute the primary antibody against viral N protein (1C7C7) in blocking solution to a final concentration of 1 μg/mL, and incubate with the cells (1 mL/well) at 37°C for 1 h.

(iv) Wash the cells 3 times with PBS.

(v) Dilute the biotinylated horse anti-mouse IgG secondary antibody in blocking solution, and incubate with the cells (1 mL/well) at 37°C for 1 h.

(vi) Prepare the ABC reagent by mixing 100 μL reagent A and 100 μL reagent B in 10 mL of PBS and incubate at 37°C for 30 min.

(vii) Wash the cells 3 times with PBS.

(viii) Incubate the cells with the ABC reagent (1 mL/well) at 37°C for 30 min.

(ix) Wash the cells 3 times with PBS.

(x) Prepare DAB substrate according to the manufacturer's instruction.

(xi) Visualize the plaques by overlaying 1 mL of the prepared DAB substrate onto the cells immediately.

(xii) Stop the staining by washing the cells with PBS after discarding the substrate solution.

Step 98: image the immunostained wells with a ChemiDoc imager. Expected results from viral plaques immunostained with the 1C7C7 N protein antibody are shown in Fig. 6b (right).

### Troubleshooting.

For a list of problems that may be encountered and their possible causes and solutions, see Table 2.

**TABLE 2:** Troubleshooting

Step	Problem	Possible reason	Possible solution
10	The concentration of empty pBeloBAC is low	Bacterial culture is inappropriate	Grow the bacteria at 37°C for at least 16 h with 250-rpm shaking in an orbital shaker
		Cell lysis is not complete	Resuspend the bacterial pellet thoroughly in solution I and let the bottle stand for 5 min after adding solution II and mixing gently
		The wash buffer is not prepared correctly	Check that ethanol has been added before using

18	Smeared bands	Plasmids have degraded	Store the pBeloBAC plasmids at −80°C
		DNase contamination of the buffer	Use a newly opened buffer

25	No colonies found after electroporation	Use of incorrect LB agar plates	Check that LB agar plates containing chloramphenicol are being used
		Contamination of residual ethanol left in the ligated DNA	Ensure that the ethanol is completely removed before resuspending the ligated DNA in nuclease-free water
		Bacteria have died after electroporation	Add SOC medium immediately after pulsing

38	Significant cell death after transfection	Low quality of the plasmid maxiprep preparation	Follow the maxiprep procedures carefully, particularly the washing step with HBC buffer
		Too few cells were used for DNA transfection	Seed a minimum of 5 × 10^5^ cells/well for transfection. Increase the no. of cells/well if needed
		Too much Lipofectamine 2000 was used	Use no more than 10 μL of Lipofectamine 2000 for each well Change the transfection medium for postinfection medium at 6 h posttransfection

65	Undesired mutations occur in pUC57-F1/Venus-2A	Mutations occurred during PCR	Always use high-fidelity DNA polymerase for PCR amplification of the DNA
		Mutations occurred during propagation of Escherichia coli	Use NEB stable competent cells for transformation

91	No fluorescence observed in transfected cells	The pBeloBAC-FL/Venus-2A plasmids are inappropriately prepared	Check that the concentration of pBeloBAC-FL/Venus-2A is adequate
		Transfection has failed	Include a plasmid expressing a fluorescent gene under a constitutive polymerase II promoter as an internal transfection control

96	No fluorescent plaques observed	The cells are killed by the semisolid medium	Overlay the semisolid medium when it is at 42°C or less
		Supernatant is overdiluted	Infect the monolayer cells with a dilution of 1:10

### Availability of materials.

pBeloBAC-FL and pBeloBAC-FL/Venus-2A for generation of rSARS-CoV-2/WT and rSARS-CoV-2/Venus-2A, respectively, are available at https://www.txbiomed.org/services-2/reverse-genetics-plasmids/. Other pBeloBAC plasmids as well as their respective rSARS-CoV-2 are also available at the same site.
