# Supplementary material for: Use of a Bacterial Artificial Chromosome to Generate Recombinant SARS-CoV-2 Expressing Robust Levels of Reporter Genes
Source: Microbiol Spectr. 2022 Nov 7;10(6):e02732-22. doi: 10.1128/spectrum.02732-22 (PMC9769592; doi:10.1128/spectrum.02732-22)
Supplement: Supplemental file 1 — Supplemental material. Download spectrum.02732-22-s0001.pdf, PDF file, 0.1 MB [file spectrum.02732-22-s0001.pdf]

Fragment 1 (F1)

CGTCTCTCATGGACATTGATTATTGACTAGTTATTAATAGTAATCAATTACGGGGTC
ATTAGTTCATAGCCCATATATGGAGTTCCGCGTTACATAACTTACGGTAAATGGCC
CGCCTGGCTGACCGCCCAACGACCCCCGCCCATTGACGTCAATAATGACGTATG
TTCCCATAGTAACGCCAATAGGGACTTTCCATTGACGTCAATGGGTGGAGTATTTA
CGGTAAACTGCCCACTTGGCAGTACATCAAGTGTATCATATGCCAAGTACGCCCC
CTATTGACGTCAATGACGGTAAATGGCCCGCCTGGCATTATGCCCAGTACATGAC
CTTATGGGACTTTCCTACTTGGCAGTACATCTACGTATTAGTCATCGCTATTACCAT
GGTGATGCGGTTTTGGCAGTACATCAATGGGCGTGGATAGCGGTTTGACTCACG
GGGATTTCCAAGTCTCCACCCCATTGACGTCAATGGGAGTTTGTTTTGGCACCA
AAATCAACGGGACTTTCCAAAATGTCGTAACAACTCCGCCCCATTGACGCAAATG
GGCGGTAGGCGTGTACGGTGGGAGGTCTATATAAGCAGAGCTCGTTTAGTGAAC
CGTATTAAAGGTTTATACCTTCCCAGGTAACAAACCAACCAACTTTCGATCTCTTG
TAGATCTGTTCTCTAAACGAACTTTAAAATCTGTGTGGCTGTCACTCGGCTGCAT
GCTTAGTGCACTCACGCAGTATAATTAATAACTAATTACTGTCGTTGACAGGACAC
GAGTAACTCGTCTATCTTCTGCAGGCTGCTTACGGTTTCGTCCGTGTTGCAGCC
GATCATCAGCACATCTAGGTTTCGTCCGGGTGTGACCGAAAGGTAAGATGGAGA
GCCTTGTCCCTGGTTTCAACGAGAAAACACACGTCCAACTCAGTTTGCCTGTTTT
ACAGGTTCGCGACGTGCTCGTACGTGGCTTTGGAGACTCCGTGGAGGAGGTCT
TATCAGAGGCACGTCAACATCTTAAAGATGGCACTTGTGGCTTAGTAGAAGTTGA
AAAAGGCGTTTTGCCTCAACTTGAACAGCCCTATGTGTTCATCAAACGTTCGGAT
GCTCGAACTGCACCTCATGGTCATGTTATGGTTGAGCTGGTAGCAGAACTCGAA
GGCATTCAGTACGGTCGTAGTGGTGAGACACTTGGTGTCCTTGTCCCTCATGTG
GGCGAAATACCAGTGGCTTACCGCAAGGTTCTTCTTCGTAAGAACGGTAATAAAG
GAGCTGGTGGCCATAGTTACGGCGCCATGTTAATTAACGCACGCGTATATTCGAA
ATGGGATCCTGCTGCAAATTTGATGAAGACGACTCTGAGCCAGTGCTCAAAGGA
GTCAAATTACATTACACATAAACGAACTTATGGATTTGTTTATGAGAATCTTCACAA
TTGGAACTGTAACTTTGAAGCAAGGTGAAATCAAGGATGCTACTCCTTCAGATTT
TGTTCGCGCTACTGCAACGATACCGATACAAGCCTCACTCCCTTTCGGATGGCTT
ATTGTTGGCGTTGCACTTCTTGCTGTTTTTCAGAGCGCTTCCAAAATCATAACCC
TCAAAAAGAGATGGCAACTAGCACTCTCCAAGGGTGTTCACTTTGTTTGCAACTT
GCTGTTGTTGTTTGTAACAGTTTACTCACACCTTTTGCTCGTTGCTGCTGGCCTT
GAAGCCCCTTTTCTCTATCTTTATGCTTTAGTCTACTTCTTGCAGAGTATAAACTTT
GTAAGAATAATAATGAGGCTTTGGCTTTGCTGGAAATGCCGTTCCAAAAACCCATT
ACTTTATGATGCCAACTATTTTCTTTGCTGGCATACTAATTGTTACGACTATTGTATA
CCTTACAATAGTGTAACTTCTTCAATTGTCATTACTTCAGGTGATGGCACAACAAG
TCCTATTTCTGAACATGACTACCAGATTGGTGGTTATACTGAAAAATGGGAATCTG
GAGTAAAAGACTGTGTTGTATTACACAGTTACTTCACTTCAGACTATTACCAGCTG
TACTCAACTCAATTGAGTACAGACACTGGTGTTGAACATGTTACCTTCTTCATCTA
CAATAAAATTGTTGATGAGCCTGAAGAACATGTCCAAATTCACACAATCGACGGT
TCATCCGGAGTTGTTAATCCAGTAATGGAACCAATTTATGATGAACCGACGACGA
CTACTAGCGTGCCTTTGTAAGCACAAGCTGATGAGTACGAACTTATGTACTCATTC
GTTTCGGAAGAGACAGGTACGTTAATAGTTAATAGCGTACTTCTTTTTCTTGCTTT

CGTGGTATTCTTGCTAGTTACACTAGCCATCCTTACTGCGCTTCGATTGTGTGCG
TACTGCTGCAATATTGTTAACGTGAGTCTTGTAAAACCTTCTTTTTACGTTTACTCT
CGTGTTAAAAATCTGAATTCTTCTAGAGTTCCTGATCTTCTGGTCTAAACGAACTA
AATATTATATTAGTTTTTCTGTTTGGAACTTTAATTTTAGCCATGGCAGATTCCAAC
GGTACTATTACCGTTGAAGAGCTTAAAAAGCTCCTTGAACAATGGAACCTAGTAAT
AGGTTTCCTATTCCTTACATGGATTTGTCTTCTACAATTTGCCTATGCCAACAGGA
ATAGGTTTTTGTATATAATTAAGTTAATTTTCCTCTGGCTGTTATGGCCAGTAACTTT
AGCTTGTTTTGTGCTTGCTGCTGTTTACAGAATAAATTGGATCACCGGTGGAATT
GCTATCGCAATGGCTTGTCTTGTAGGCTTGATGTGGCTCAGCTACTTCATTGCTT
CTTTCAGACTGTTTGCGCGTACGCGATCCATGTGGTCATTCAATCCAGAAACTAA
CATTCTTCTCAACGTGCCACTCCATGGCACTATTCTGACCAGACCGCTTCTAGAA
AGTGAACTCGTAATCGGAGCTGTGATCCTTCGTGGACATCTTCGTATTGCTGGAC
ACCATCTAGGACGCTGTGACATCAAGGACCTGCCTAAAGAAATCACTGTTGCTAC
ATCACGAACGCTTTCTTATTACAAATTGGGAGCTTCGCAGCGTGTAGCAGGTGAC
TCAGGTTTTGCTGCATACAGTCGCTACAGGATTGGCAACTATAAATTAAACACAGA
CCATTCCAGTAGCAGTGACAATATTGCTTTGCTTGTACAGTAAGTGACAACAGAT
GTTTCATCTCGTTGACTTTCAGGTTACTATAGCAGAGATATTACTAATTATTATGAG
GACTTTTAAAGTTTCCATTTGGAATCTTGATTACATCATAAACCTCATAATTAAAAAT
TTATCTAAGTCACTAACTGAGAATAAATATTCTCAATTAGATGAAGAGCAACCAATG
GAGATTGATTAAACGAACATGAAAATTATTCTTTTCTTGGCACTGATAACACTCGC
TACTTGTGAGCTTTATCACTACCAAGAGTGTGTTAGAGGTACAACAGTACTTTTAA
AAGAACCTTGCTCTTCTGGAACATACGAGGGCAATTCACCATTTCATCCTCTAGC
TGATAACAAATTTGCACTGACTTGCTTTAGCACTCAATTTGCTTTTGCTTGTCCTG
ACGGCGTAAAACACGTCTATCAGTTACGTGCCAGATCAGTTTCACCTAAACTGTT
CATCAGACAAGAGGAAGTTCAAGAACTTTACTCTCCAATTTTTCTTATTGTTGCGG
CAATAGTGTTTATAACACTTTGCTTCACACTCAAAAGAAAGACAGAATGATTGAAC
TTTCATTAATTGACTTCTATTTGTGCTTTTTAGCCTTTCTGCTATTCCTTGTTTTAAT
TATGCTTATTATCTTTTGGTTCTCACTTGAACTGCAAGATCATAATGAAACTTGTCA
CGCCTAAACGAACATGAAATTTCTTGTTTTCTTAGGAATCATCACAACTGTAGCTG
CATTTCACCAAGAATGTAGTTTACAGTCATGTACTCAACATCAACCATATGTAGTTG
ATGACCCGTGTCCTATTCACTTCTATTCTAAATGGTATATTAGAGTAGGAGCTAGAA
AATCAGCACCTTTAATTGAATTGTGCGTGGATGAGGCTGGTTCTAAATCACCCATT
CAGTACATCGATATCGGTAATTATACAGTTTCCTGTTCACCTTTTACAATTAATTGC
CAGGAACCTAAATTGGGTAGTCTTGTAGTGCGTTGTTCGTTCTATGAAGACTTTTT
AGAGTATCATGACGTTCGTGTTGTTTTAGATTTCATCTAAACGAACAAACTAAAAT
GTCTGATAATGGACCCCAAAATCAGCGAAATGCACCCCGCATTACGTTTGGTGGA
CCCTCAGATTCAACTGGCAGTAACCAGAATGGAGAACGCAGTGGGGCGCGATC
AAAACAACGTCGGCCCCAAGGTTTACCCAATAATACTGCGTCTTGGTTCACCGCT
CTCACTCAACATGGCAAGGAAGACCTTAAATTCCCTCGAGGACAAGGCGTTCCA
ATTAACACCAATAGCAGTCCAGATGACCAAATTGGCTACTACCGAAGAGCTACCA
GACGAATTCGTGGTGGTGACGGTAAAATGAAAGATCTCAGTCCAAGATGGTATTT
CTACTACCTAGGAACTGGGCCAGAAGCTGGACTTCCCTATGGTGCTAACAAAGA
CGGCATCATATGGGTTGCAACTGAGGGAGCCTTGAATACACCAAAAGATCACATT
GGCACCCGCAATCCTGCTAACAATGCTGCAATCGTGCTACAACTTCCTCAAGGA

ACAACATTGCCAAAAGGCTTCTACGCAGAAGGGAGCAGAGGCGGCAGTCAAGC
CTCTTCTCGTTCCTCATCACGTAGTCGCAACAGTTCAAGAAATTCAACTCCAGGC
AGCAGTAGGGGAACTTCTCCTGCTAGAATGGCTGGCAATGGCGGTGATGCTGCT
CTTGCTTTGCTGCTGCTTGACAGATTGAACCAGCTTGAGAGCAAAATGTCTGGTA
AAGGCCAACAACAACAAGGCCAAACTGTCACTAAGAAATCTGCTGCTGAGGCTT
CTAAGAAGCCTCGGCAAAAACGTACTGCCACTAAAGCATACAATGTAACACAAGC
TTTCGGCAGACGTGGTCCAGAACAAACCCAAGGAAATTTTGGGGACCAGGAACT
AATCAGACAAGGAACTGATTACAAACATTGGCCGCAAATTGCACAATTTGCCCCC
AGCGCTTCAGCGTTCTTCGGAATGTCGCGCATTGGCATGGAAGTCACACCTTCG
GGAACGTGGTTGACCTACACAGGTGCCATCAAATTGGATGACAAAGATCCAAATT
TCAAAGATCAAGTCATTTTGCTGAATAAGCATATTGACGCATACAAAACATTCCCA
CCAACAGAGCCTAAAAAGGACAAAAAGAAGAAGGCTGATGAAACTCAAGCCTTA
CCGCAGAGACAGAAGAAACAGCAAACTGTGACTCTTCTTCCTGCTGCAGATTTG
GATGATTTCTCCAAACAATTGCAACAATCCATGAGCAGTGCTGACTCAACTCAGG
CCTAAACTCATGCAGACCACACAAGGCAGATGGGCTATATAAACGTTTTCGCTTT
TCCGTTTACGATATATAGTCTACTCTTGTGCAGAATGAATTCTCGTAACTACATAGC
ACAAGTAGATGTAGTTAACTTTAATCTCACATAGCAATCTTTAATCAGTGTGTAACA
TTAGGGAGGACTTGAAAGAGCCACCACATTTTCACCGAGGCCACGCGGAGTAC
GATCGAGTGTACAGTGAACAATGCTAGGGAGAGCTGCCTATATGGAAGAGCCCT
AATGTGTAAAATTAATTTTAGTAGTGCTATCCCCATGTGATTTTAATAGCTTCTTAGG
AGAATGACAAAAAAAAAAAAAAAAAAAAAAAAAAAAAAAAAA*gggtcggcatggcatctccac*
ctcctcgcggtccgacctgggcatccgaaggaggacgtcgtccactcggatggctaagggagagctcggatcgatc
*cgcctcga*ctgtgccttctagttgccagccatctgttgtttgcccctcccccgtgccttccttgaccctggaaggtgccactc
ccactgtcctttcctaataaaatgaggaaattgcatcgcattgtctgagtaggtgtcattctattctggggggtggggtggg
gcaggacagcaagggggaggattgggaagacaatagcaggcatgctggggaAGCTAGAGACG

- The underlined sequences in capital (CGTCTCT and AGAGACG) indicate the BsmBI restriction site used to generate overhangs that are compatible with PciI (ACATGT) and HindIII (AAGCTT) (**Figure 1**).
- The sequence highlighted in red represents the cytomegalovirus (CMV) promoter.
- The sequences highlighted in yellow represent unique KasI (GGCGCC), PacI (TTAATTAA), MluI (ACGCGT), BstBI (TTCGAA), and BamHI (GGATCC) restriction sites use to clone F2-F5 (**Figure 1**).
- The single nucleotide highlighted in green indicates a silent mutation introduced to remove a MluI (ACGCGT) restriction site present in the viral membrane (M) gene to clone F3 (**Figure 1**). This single nucleotide was also used as a genetic tag to distinguish the rSARS-CoV-2 from the natural SARS-CoV-2 isolate.
- The sequence shown in lower case italic font indicates the hepatitis delta virus (HDV) ribozyme (Rz) sequence.
- The lower case underlined sequence represents the bovine growth hormone (bGH) polyadenylation signal.

Fragment 2 (F2)

GGCGCCGATCTAAAGTCATTTGACTTAGGCGACGAGCTTGGCACTGATCCTTATGAAGATTTTCAAGAAAACTGGAACACTAAACATAGCAGTGGTGTTACCCGTGAACTCATGCGTGAGCTTAACGGAGGGGCATACACTCGCTATGTCGATAACAACTTCTGTGGCCCTGATGGCTACCCTCTTGAGTGCATTAAAGACCTTCTAGCACGTGCTGGTAAAGCTTCATGCACTTTGTCCGAACAACTGGACTTTATTGACACTAAGAGGGGTGTATACTGCTGCCGTGAACATGAGCATGAAATTGCTTGGTACACGGAACGTTCTGAAAAGAGCTATGAATTGCAGACACCTTTTGAAATTAAATTGGCAAAGAAATTTGACACCTTCAATGGGGAATGTCCAAATTTTGTATTTCCCTTAAATTCCATAATCAAGACTATTCAACCAAGGGTTGAAAAGAAAAAGCTTGATGGCTTTATGGGTAGAATTCGATCTGTCTATCCAGTTGCGTCACCAAATGAATGCAACCAAATGTGCCTTTCAACTCTCATGAAGTGTGATCATTGTGGTGAAACTTCATGGCAGACGGGCGATTTTGTTAAAGCCACTTGCGAATTTTGTGGCACTGAGAATTTGACTAAAGAAGGTGCCACTACTTGTGGTTACTTACCCCAAAATGCTGTTGTTAAAATTTATTGTCCAGCATGTCACAATTCAGAAGTAGGACCTGAGCATAGTCTTGCCGAATACCATAATGAATCTGGCTTGAAAACCATTCTTCGTAAGGGTGGTCGCACTATTGCCTTTGGAGGCTGTGTGTTCTCTTATGTTGGTTGCCATAACAAGTGTGCCTATTGGGTTCCACGTGCTAGCGCTAACATAGGTTGTAACCATACAGGTGTTGTTGGAGAAGGTTCCGAAGGTCTTAATGACAACCTTCTTGAAATACTCCAAAAAGAGAAAGTCAACATCAATATTGTTGGTGACTTTAAACTTAATGAAGAGATCGCCATTATTTTGGCATCTTTTTCTGCTTCCACAAGTGCTTTTGTGGAAACTGTGAAAGGTTTGGATTATAAAGCATTCAAACAAATTGTTGAATCCTGTGGTAATTTTAAAGTTACAAAAGGAAAAGCTAAAAAAGGTGCCTGGAATATTGGTGAACAGAAATCAATACTGAGTCCTCTTTATGCATTTGCATCAGAGGCTGCTCGTGTTGTACGATCAATTTTCTCCCGCACTCTTGAAACTGCTCAAAATTCTGTGCGTGTTTTACAGAAGGCCGCTATAACAATACTAGATGGAATTTCACAGTATTCACTGAGACTCATTGATGCTATGATGTTCACATCTGATTTGGCTACTAACAATCTAGTTGTAATGGCCTACATTACAGGTGGTGTTGTTCAGTTGACTTCGCAGTGGCTAACTAACATCTTTGGCACTGTTTATGAAAAACTCAAACCCGTCCTTGATTGGCTTGAAGAGAAGTTTAAGGAAGGTGTAGAGTTTCTTAGAGACGGTTGGGAAATTGTTAAATTTATCTCAACCTGTGCTTGTGAAATTGTCGGTGGACAAATTGTCACCTGTGCAAAGGAAATTAAGGAGAGTGTTCAGACATTCTTTAAGCTTGTAAATAAATTTTTGGCTTTGTGTGCTGACTCTATCATTATTGGTGGAGCTAAACTTAAAGCCTTGAATTTAGGTGAAACATTTGTCACGCACTCAAAGGGATTGTACAGAAAGTGTGTTAAATCCAGAGAAGAAACTGGCCTACTCATGCCTCTAAAAGCCCCAAAAGAAATTATCTTCTTAGAGGGAGAAACACTTCCCACAGAAGTGTTAACAGAGGAAGTTGTCTTGAAAACTGGTGATTTACAACCATTAGAACAACCTACTAGTGAAGCTGTTGAAGCTCCATTGGTTGGTACACCAGTTTGTATTAACGGGCTTATGTTGCTCGAAATCAAAGACACAGAAAAGTACTGTGCCCTTGCACCTAATATGATGGTAACAAACAATACCTTCACACTCAAAGGCGGTGCACCAACAAAGGTTACTTTTGGTGATGACACTGTGATAGAAGTGCAAGGTTACAAGAGTGTGAATATCACTTTTGAACTTGATGAAAGGATTGATAAAGTACTTAATGAGAAGTGCTCTGCCTATACAGTTGAACTCGGTACAGAAGTAAATGAGTTCGCCTGTGTTGTGGCAGATGCTGTCATAAAAACTTTGCAACCAGTATCTGAATTACTTACACCACTGGGCATTGATTTAGATGAGTGGAGTATGGCTACATACTACTTATTTGATGAGTCTG

GTGAGTTTAAATTGGCTTCACATATGTATTGTTCTTTCTACCCTCCAGATGAGGAT
GAAGAAGAAGGTGATTGTGAAGAAGAAGAGTTTGAGCCATCAACTCAATATGAGT
ATGGTACTGAAGATGATTACCAAGGTAAACCTTTGGAATTTGGTGCCACTTCTGC
TGCTCTTCAACCTGAAGAAGAGCAAGAAGAAGATTGGTTAGATGATGATAGTCAA
CAAACTGTTGGTCAACAAGACGGCAGTGAGGACAATCAGACAACTACTATTCAAA
CAATTGTTGAGGTTCAACCTCAATTAGAGATGGAACTTACACCAGTTGTTCAGAC
TATTGAAGTGAATAGTTTTAGTGGTTATTTAAAACTTACTGACAATGTATACATTAAA
AATGCAGACATTGTGGAAGAAGCTAAAAAGGTAAAACCAACAGTGGTTGTTAATG
CAGCCAATGTTTACCTTAAACATGGAGGAGGTGTTGCAGGAGCCTTAAATAAGGC
TACTAACAATGCCATGCAAGTTGAATCTGATGATTACATAGCTACTAATGGACCAC
TTAAAGTGGGTGGTAGTTGTGTTTTAAGCGGACACAATCTTGCTAAACACTGTCT
TCATGTTGTCGGCCCAAATGTTAACAAAGGTGAAGACATTCAACTTCTTAAGAGT
GCTTATGAAAATTTTAATCAGCACGAAGTTCTACTTGCACCATTATTATCAGCTGGT
ATTTTTGGTGCTGACCCTATACATTCTTTAAGAGTTTGTGTAGATACTGTTCGCAC
AAATGTCTACTTAGCTGTCTTTGATAAAAATCTCTATGACAAACTTGTTTCAAGCTT
TTTGGAAATGAAGAGTGAAAAGCAAGTTGAACAAAAGATCGCTGAGATTCCTAAA
GAGGAAGTTAAGCCATTTATAACTGAAAGTAAACCTTCAGTTGAACAGAGAAAAC
AAGATGATAAGAAAATCAAAGCTTGTGTTGAAGAAGTTACAACAACTCTGGAAGA
AACTAAGTTCCTCACAGAAAACTTGTTACTTTATATTGACATTAATGGCAATCTTCA
TCCAGATTCTGCCACTCTTGTTAGTGACATTGACATCACTTTCTTAAAGAAAGATG
CTCCATATATAGTGGGTGATGTTGTTCAAGAGGGTGTTTTAACTGCTGTGGTTATA
CCTACTAAAAAGGCTGGTGGCACTACTGAAATGCTAGCGAAAGCTTTGAGAAAA
GTGCCAACAGACAATTATATAACCACTTACCCGGGTCAGGGTTTAAATGGTTACA
CTGTAGAGGAGGCAAAGACAGTGCTTAAAAAGTGTAAAAGTGCCTTTTACATTCT
ACCATCTATTATCTCTAATGAGAAGCAAGAAATTCTTGGAACTGTTTCTTGGAATTT
GCGAGAAATGCTTGCACATGCAGAAGAAACACGCAAATTAATGCCTGTCTGTGT
GGAAACTAAAGCCATAGTTTCAACTATACAGCGTAAATATAAGGGTATTAAAATACA
AGAGGGTGTGGTTGATTATGGTGCTAGATTTTACTTTTACACCAGTAAAACAACTG
TAGCGTCACTTATCAACACACTTAACGATCTAAATGAAACTCTTGTTACAATGCCA
CTTGGCTATGTAACACATGGCTTAAATTTGGAAGAAGCTGCTCGGTATATGAGATC
TCTCAAAGTGCCAGCTACAGTTTCTGTTTCTTCACCTGATGCTGTTACAGCGTATA
ATGGTTATCTTACTTCTTCTTCTAAAACACCTGAAGAACATTTTATTGAAACCATCT
CACTTGCTGGTTCCTATAAAGATTGGTCCTATTCTGGACAATCTACACAACTAGGT
ATAGAATTTCTTAAGAGAGGTGATAAAAGTGTATATTACACTAGTAATCCTACCACA
TTCCACCTAGATGGTGAAGTTATCACCTTTGACAATCTTAAGACACTTCTTTCTTT
GAGAGAAGTGAGGACTATTAAGGTGTTTACAACAGTAGACAACATTAACCTCCAC
ACGCAAGTTGTGGACATGTCAATGACATATGGACAACAGTTTGGTCCAACTTATT
TGGATGGAGCTGATGTTACTAAAATAAAACCTCATAATTCACATGAAGGTAAAACA
TTTTATGTTTTACCTAATGATGACACTCTACGTGTTGAGGCTTTTGAGTACTACCA
CACAACTGATCCTAGTTTTCTGGGTAGGTACATGTCAGCATTAAATCACACTAAAA
AGTGGAAATACCCACAAGTTAATGGTTTAACTTCTATTAAATGGGCAGATAACAAC
TGTTATCTTGCCACTGCATTGTTAACACTCCAACAAATAGAGTTGAAGTTTAATCC
ACCTGCTCTACAAGATGCTTATTACAGAGCAAGGGCTGGTGAAGCTGCTAACTTT
TGTGCACTTATCTTAGCCTACTGTAATAAGACAGTAGGTGAGTTAGGTGATGTTAG

AGAAACAATGAGTTACTTGTTTCAACATGCCAATTTAGATTCTTGCAAAAGAGTCT
TGAACGTGGTGTGTAAAACTTGTGGACAACAGCAGACAACCCTTAAGGGTGTAG
AAGCTGTTATGTACATGGGCACACTTTCTTATGAACAATTTAAGAAAGGTGTTCAG
ATACCTTGTACGTGTGGTAAACAAGCTACAAAATATCTAGTACAACAGGAGTCACC
TTTTGTTATGATGTCAGCACCACCTGCTCAGTATGAACTTAAGCATGGTACATTTA
CTTGTGCTAGTGAGTACACTGGTAATTACCAGTGTGGTCACTATAAACATATAACT
TCTAAAGAAACTTTGTATTGCATAGACGGTGCTTTACTTACAAAGTCCTCAGAATA
CAAAGGTCCTATTACGGATGTTTTCTACAAAGAAAACAGTTACACAACAACCATAA
AACCAGTTACTTATAAATTGGATGGTGTTGTTTGTACAGAAATTGACCCTAAGTTG
GACAATTATTATAAGAAAGACAATTCTTATTTCACAGAGCAACCAATTGATCTTGTA
CCAAACCAACCATATCCAAACGCAAGCTTCGATAATTTTAAGTTTGTATGTGATAAT
ATCAAATTTGCTGATGATTTAAACCAGTTAACTGGTTATAAGAAACCTGCTTCAAG
AGAGCTTAAAGTTACATTTTTCCCTGACTTAAATGGTGATGTGGTGGCTATTGATT
ATAAACACTACACACCCTCTTTTAAGAAAGGAGCTAAATTGTTACATAAACCTATTG
TTTGGCATGTTAACAATGCAACTAATAAAGCCACGTATAAACCAAATACCTGGTGT
ATACGTTGTCTTTGGAGCACAAAACCAGTTGAAACATCAAATTCGTTTGATGTACT
GAAGTCAGAGGACGCGCAGGGAATGGATAATCTTGCCTGCGAAGATCTAAAACC
AGTCTCTGAAGAAGTAGTGGAAAATCCTACCATACAGAAAGACGTTCTTGAGTGT
AATGTGAAAACTACCGAAGTTGTAGGAGACATTATACTTAAACCAGCAAATAATAG
TTTAAAAATTACAGAAGAGGTTGGCCACACAGATCTAATGGCTGCTTATGTAGACA
ATTCTAGTCTTACTATTAAGAAACCTAATGAATTATCTAGAGTATTAGGTTTGAAAAC
CCTTGCTACTCATGGTTTAGCTGCTGTTAATAGTGTCCCTTGGGATACTATAGCTA
ATTATGCTAAGCCTTTTCTTAACAAAGTTGTTAGTACAACTACTAACATAGTTACAC
GGTGTTTAAACCGTGTTTGTACTAATTATATGCCTTATTTCTTTACTTTATTGCTACA
ATTGTGTACTTTTACTAGAAGTACAAATTCTAGAATTAAAGCATCTATGCCGACTAC
TATAGCAAAGAATACTGTTAAGAGTGTCGGTAAATTTTGTCTAGAGGCTTCATTTAA
TTATTTGAAGTCACCTAATTTTTCTAAACTGATAAATATTATAATTTGGTTTTTACTAT
TAAGTGTTTGCCTAGGTTCTTTAATCTACTCAACCGCTGCTTTAGGTGTTTTAATG
TCTAATTTAGGCATGCCTTCTTACTGTACTGGTTACAGAGAAGGCTATTTGAACTC
TACTAATGTCACTATTGCAACCTACTGTACTGGTTCTATACCTTGTAGTGTTTGTCT
TAGTGGTTTAGATTCTTTAGACACCTATCCTTCTTTAGAAACTATACAAATTACCATT
TCATCTTTTAAATGGGATTTAACTGCTTTTGGCTTAGTTGCAGAGTGGTTTTTGGC
ATATATTCTTTTCACTAGGTTTTTCTATGTACTTGGATTGGCTGCAATCATGCAATT
GTTTTTCAGCTATTTTGCAGTACATTTTATTAGTAATTCTTGGCTTATGTGGTTAATA
ATTAATCTTGTACAAATGGCCCCGATTTCAGCTATGGTTAGAATGTACATCTTCTTT
GCATCATTTTATTATGTATGGAAAAGTTATGTGCATGTTGTAGACGGTTGTAATTCA
TCAACTTGTATGATGTGTTACAAACGTAATAGAGCAACAAGAGTCGAATGTACAAC
TATTGTTAATGGTGTTAGAAGGTCCTTTTATGTCTATGCTAATGGAGGTAAAGGCT
TTTGCAAACTACACAATTGGAATTGTGTTAATTGTGATACATTCTGTGCTGGTAGT
ACATTTATTAGTGATGAAGTTGCGAGAGACTTGTCACTACAGTTTAAAAGACCAAT
AAATCCTACTGACCAGTCTTCTTACATCGTTGATAGTGTTACAGTGAAGAATGGTT
CCATCCATCTTTACTTTGATAAAGCTGGTCAAAAGACTTATGAAAGACATTCTCTC
TCTCATTTTGTTAACTTAGACAACCTGAGAGCTAATAACACTAAAGGTTCATTGCC
TATTAATGTTATAGTTTTTGATGGTAAATCAAAATGTGAAGAATCATCTGCAAAATCA

GCGTCTGTTTACTACAGTCAGCTTATGTGTCAACCTATACTGTTACTAGATCAGGC
ATTAGTGTCTGATGTTGGTGATAGTGCGGAAGTTGCAGTTAAAATGTTTGATGCTT
ACGTTAATACGTTTTCATCAACTTTTAACGTACCAATGGAAAAACTCAAAACACTA
GTTGCAACTGCAGAAGCTGAACTTGCAAAGAATGTGTCCTTAGACAATGTCTTAT
CTACTTTTATTTCAGCAGCTCGGCAAGGGTTTGTTGATTCAGATGTAGAAACTAAA
GATGTTGTTGAATGTCTTAAATTGTCACATCAATCTGACATAGAAGTTACTGGCGA
TAGTTGTAATAACTATATGCTCACCTATAACAAAGTTGAAAACATGACACCCCGTG
ACCTTGGTGCTTGTATTGACTGTAGTGCGCGTCATATTAATGCGCAGGTAGCAAA
AAGTCACAACATTGCTTTGATATGGAACGTTAAAGATTTCATGTCATTGTCTGAAC
AACTACGAAAACAAATACGTAGTGCTGCTAAAAAGAATAACTTACCTTTTAAGTTG
ACATGTGCAACTACTAGACAAGTTGTTAATGTTGTAACAACAAAGATAGCACTTAA
GGGTGGTAAAATTGTTAATAATTGGTTGAAGCAGTTAATTAA

- The sequences highlighted in yellow indicate unique Kas I (GGCGCC) and PacI (TTAATTAA) restriction sites used to clone F2 (**Figure 1**).

Fragment 3 (F3)

TTAATTAAAGTTACACTTGTGTTCCTTTTTGTTGCTGCTATTTTCTATTTAATAACAC
CTGTTCATGTCATGTCTAAACATACTGACTTTTCAAGTGAAATCATAGGATACAAG
GCTATTGATGGTGGTGTCACTCGTGACATAGCATCTACAGATACTTGTTTTGCTAA
CAAACATGCTGATTTTGACACATGGTTTAGTCAGCGTGGTGGTAGTTATACTAATG
ACAAAGCTTGCCCATTGATTGCTGCAGTCATAACAAGAGAAGTGGGTTTTGTCGT
GCCTGGTTTGCCTGGCACGATATTACGCACAACTAATGGTGACTTTTTGCATTTC
TTACCTAGAGTTTTTAGTGCAGTTGGTAACATCTGTTACACACCATCAAAACTTATA
GAGTACACTGACTTTGCAACATCAGCTTGTGTTTTGGCTGCTGAATGTACAATTTT
TAAAGATGCTTCTGGTAAGCCAGTACCATATTGTTATGATACCAATGTACTAGAAG
GTTCTGTTGCTTATGAAAGTTTACGCCCTGACACACGTTATGTGCTCATGGATGG
CTCTATTATTCAATTTCCTAACACCTACCTTGAAGGTTCTGTTAGAGTGGTAACAA
CTTTTGATTCTGAGTACTGTAGGCACGGCACTTGTGAAAGATCAGAAGCTGGTGT
TTGTGTATCTACTAGTGGTAGATGGGTACTTAACAATGATTATTACAGATCTTTACC
AGGAGTTTTCTGTGGTGTAGATGCTGTAAATTTACTTACTAATATGTTTACACCACT
AATTCAACCTATTGGTGCTTTGGACATATCAGCATCTATAGTAGCTGGTGGTATTG
TAGCTATCGTAGTAACATGCCTTGCCTACTATTTTATGAGGTTTAGAAGAGCTTTTG
GTGAATACAGTCATGTAGTTGCCTTTAATACTTTACTATTCCTTATGTCATTCACTG
TACTCTGTTTAACACCAGTTTACTCATTCTTACCTGGTGTTTATTCTGTTATTTACTT
GTACTTGACATTTTATCTTACTAATGATGTTTCTTTTTTAGCACATATTCAGTGGATG
GTTATGTTCACACCTTTAGTACCTTTCTGGATAACAATTGCTTATATCATTTGTATTT
CCACAAAGCATTTCTATTGGTTCTTTAGTAATTACCTAAAGAGACGTGTAGTCTTTA
ATGGTGTTTCCTTTAGTACTTTTGAAGAAGCTGCGCTGTGCACCTTTTTGTTAAAT
AAAGAAATGTATCTAAAGTTGCGTAGTGATGTGCTATTACCTCTTACGCAATATAAT
AGATACTTAGCTCTTTATAATAAGTACAAGTATTTTAGTGGAGCAATGGATACAACT
AGCTACAGAGAAGCTGCTTGTTGTCATCTCGCAAAGGCTCTCAATGACTTCAGTA
ACTCAGGTTCTGATGTTCTTTACCAACCACCACAAACCTCTATCACCTCAGCTGT
TTTGCAGAGTGGTTTTAGAAAAATGGCATTCCCATCTGGTAAAGTTGAGGGTTGT
ATGGTACAAGTAACTTGTGGTACAACTACACTTAACGGTCTTTGGCTTGATGACG
TAGTTTACTGTCCAAGACATGTGATCTGCACCTCTGAAGACATGCTTAACCCTAAT
TATGAAGATTTACTCATTCGTAAGTCTAATCATAATTTCTTGGTACAGGCTGGTAAT
GTTCAACTCAGGGTTATTGGACATTCTATGCAAAATTGTGTACTTAAGCTTAAGGT
TGATACAGCCAATCCTAAGACACCTAAGTATAAGTTTGTTCGCATTCAACCAGGAC
AGACTTTTTCAGTGTTAGCTTGTTACAATGGTTCACCATCTGGTGTTTACCAATGT
GCTATGAGGCCCAATTTCACTATTAAGGGTTCATTCCTTAATGGTTCATGTGGTAG
TGTTGGTTTTAACATAGATTATGACTGTGTCTCTTTTTGTTACATGCACCATATGGA
ATTACCAACTGGAGTTCATGCTGGCACAGACTTAGAAGGTAACTTTTATGGACCT
TTTGTTGACAGGCAAACAGCACAAGCAGCTGGTACGGACACAACTATTACAGTTA
ATGTTTTAGCTTGGTTGTACGCTGCTGTTATAAATGGAGACAGGTGGTTTCTCAAT
CGATTTACCACAACTCTTAATGACTTTAACCTTGTGGCTATGAAGTACAATTATGAA
CCTCTAACACAAGACCATGTTGACATACTAGGACCTCTTTCTGCTCAAACTGGAA
TTGCCGTTTTAGATATGTGTGCTTCATTAAAAGAATTACTGCAAAATGGTATGAATG
GACGTACCATATTGGGTAGTGCTTTATTAGAAGATGAATTTACACCTTTTGATGTTG

TTAGACAATGCTCAGGTGTTACTTTCCAAAGTGCAGTGAAAAGAACAATCAAGGG
TACACACCACTGGTTGTTACTCACAATTTTGACTTCACTTTTAGTTTTAGTCCAGA
GTACTCAATGGTCTTTGTTCTTTTTTTTGTATGAAAATGCCTTTTTACCTTTTGCTAT
GGGTATTATTGCTATGTCTGCTTTTGCAATGATGTTTGTCAAACATAAGCATGCATT
TCTCTGTTTGTTTTTGTTACCTTCTCTTGCCACTGTAGCTTATTTTAATATGGTCTAT
ATGCCTGCTAGTTGGGTGATGCGTATTATGACATGGTTGGATATGGTTGATACTAG
TTTGTCTGGTTTTAAGCTAAAAGACTGTGTTATGTATGCATCAGCTGTAGTGTTAC
TAATCCTTATGACAGCAAGAACTGTGTATGATGATGGTGCTAGGAGAGTGTGGAC
ACTTATGAATGTCTTGACACTCGTTTATAAAGTTTATTATGGTAATGCTTTAGATCAA
GCCATTTCCATGTGGGCTCTTATAATCTCTGTTACTTCTAACTACTCAGGTGTAGT
TACAACTGTCATGTTTTTGGCCAGAGGTATTGTTTTTATGTGTGTTGAGTATTGCC
CTATTTTCTTCATAACTGGTAATACACTTCAGTGTATAATGCTAGTTTATTGTTTCTT
AGGCTATTTTTGTACTTGTTACTTTGGCCTCTTTTGTTTACTCAACCGCTACTTTAG
ACTGACTCTTGGTGTTTATGATTACTTAGTTTCTACACAGGAGTTTAGATATATGAA
TTCACAGGGACTACTCCCACCCAAGAATAGCATAGATGCCTTCAAACTCAACATT
AAATTGTTGGGTGTTGGTGGCAAACCTTGTATCAAAGTAGCCACTGTACAGTCTA
AAATGTCAGATGTAAAGTGCACATCAGTAGTCTTACTCTCAGTTTTGCAACAACTC
AGAGTAGAATCATCATCTAAATTGTGGGCTCAATGTGTCCAGTTACACAATGACAT
TCTCTTAGCTAAAGATACTACTGAAGCCTTTGAAAAAATGGTTTCACTACTTTCTG
TTTTGCTTTCCATGCAGGGTGCTGTAGACATAAACAAGCTTTGTGAAGAAATGCT
GGACAACAGGGCAACCTTACAAGCTATAGCCTCAGAGTTTAGTTCCCTTCCATCA
TATGCAGCTTTTGCTACTGCTCAAGAAGCTTATGAGCAGGCTGTTGCTAATGGTG
ATTCTGAAGTTGTTCTTAAAAAGTTGAAGAAGTCTTTGAATGTGGCTAAATCTGAA
TTTGACCGTGATGCAGCCATGCAACGTAAGTTGGAAAAGATGGCTGATCAAGCTA
TGACCCAAATGTATAAACAGGCTAGATCTGAGGACAAGAGGGCAAAAGTTACTAG
TGCTATGCAGACAATGCTTTTCACTATGCTTAGAAAGTTGGATAATGATGCACTCA
ACAACATTATCAACAATGCAAGAGATGGTTGTGTTCCCTTGAACATAATACCTCTT
ACAACAGCAGCCAAACTAATGGTTGTCATACCAGACTATAACACATATAAAAATAC
GTGTGATGGTACAACATTTACTTATGCATCAGCATTGTGGGAAATCCAACAGGTT
GTAGATGCAGATAGTAAAATTGTTCAACTTAGTGAAATTAGTATGGACAATTCACCT
AATTTAGCATGGCCTCTTATTGTAACAGCTTTAAGGGCCAATTCTGCTGTCAAATT
ACAGAATAATGAGCTTAGTCCTGTTGCACTACGACAGATGTCTTGTGCTGCCGGT
ACTACACAAACTGCTTGCACTGATGACAATGCGTTAGCTTACTACAACACAACAA
AGGGAGGTAGGTTTGTACTTGCACTGTTATCCGATTTACAGGATTTGAAATGGGC
TAGATTCCCTAAGAGTGATGGAACTGGTACTATCTATACAGAACTGGAACCACCTT
GTAGGTTTGTTACAGACACACCTAAAGGTCCTAAAGTGAAGTATTTATACTTTATTA
AAGGATTAAACAACCTAAATAGAGGTATGGTACTTGGTAGTTTAGCTGCCACAGTA
CGTCTACAAGCTGGTAATGCAACAGAAGTGCCTGCCAATTCAACTGTATTATCTTT
CTGTGCTTTTGCTGTAGATGCTGCTAAAGCTTACAAAGATTATCTAGCTAGTGGG
GGACAACCAATCACTAATTGTGTTAAGATGTTGTGTACACACACTGGTACTGGTC
AGGCAATAACAGTTACACCGGAAGCCAATATGGATCAAGAATCCTTTGGTGGTGC
ATCGTGTTGTCTGTACTGCCGTTGCCACATAGATCATCCAAATCCTAAAGGATTTT
GTGACTTAAAAGGTAAGTATGTACAAATACCTACAACTTGTGCTAATGACCCTGTG
GGTTTTACACTTAAAAACACAGTCTGTACCGTCTGCGGTATGTGGAAAGGTTATG

GCTGTAGTTGTGATCAACTCCGCGAACCCATGCTTCAGTCAGCTGATGCACAATC
GTTTTTAAACGGGTTTGCGGTGTAAGTGCAGCCCGTCTTACACCGTGCGGCACA
GGCACTAGTACTGATGTCGTATACAGGGCTTTTGACATCTACAATGATAAAGTAGC
TGGTTTTGCTAAATTCCTAAAAACTAATTGTTGTCGCTTCCAAGAAAAGGACGAAG
ATGACAATTTAATTGATTCTTACTTTGTAGTTAAGAGACACACTTTCTCTAACTACC
AACATGAAGAAACAATTTATAATTTACTTAAGGATTGTCCAGCTGTTGCTAAACATG
ACTTCTTTAAGTTTAGAATAGACGGTGACATGGTACCACATATATCACGTCAACGT
CTTACTAAATACACAATGGCAGACCTCGTCTATGCTTTAAGGCATTTTGATGAAGG
TAATTGTGACACATTAAAAGAAATACTTGTCACATACAATTGTTGTGATGATGATTA
TTTCAATAAAAAGGACTGGTATGATTTTGTAGAAAACCCAGATATATTACGCGT

- The sequences highlighted in yellow indicate unique PacI (TTAATTAA) and MluI (ACGCGT) restriction sites used to clone F3 (**Figure 1**).

Fragment 4 (F4)

ACGCGTATACGCCAACTTAGGTGAACGTGTACGCCAAGCTTTGTTAAAAACAGTA
CAATTCTGTGATGCCATGCGAAATGCTGGTATTGTTGGTGTACTGACATTAGATAA
TCAAGATCTCAATGGTAACTGGTATGATTTCGGTGATTTCATACAAACCACGCCAG
GTAGTGGAGTTCCTGTTGTAGATTCTTATTATTCATTGTTAATGCCTATATTAACCTT
GACCAGGGCTTTAACTGCAGAGTCACATGTTGACACTGACTTAACAAAGCCTTAC
ATTAAGTGGGATTTGTTAAAATATGACTTCACGGAAGAGAGGTTAAAACTCTTTGA
CCGTTATTTTAAATATTGGGATCAGACATACCACCCAAATTGTGTTAACTGTTTGGA
TGACAGATGCATTCTGCATTGTGCAAACTTTAATGTTTTATTCTCTACAGTGTTCC
CACCTACAAGTTTTGGACCACTAGTGAGAAAAATATTTGTTGATGGTGTTCCATTT
GTAGTTTCAACTGGATACCACTTCAGAGAGCTAGGTGTTGTACATAATCAGGATG
TAAACTTACATAGCTCTAGACTTAGTTTTAAGGAATTACTTGTGTATGCTGCTGACC
CTGCTATGCACGCTGCTTCTGGTAATCTATTACTAGATAAACGCACTACGTGCTTT
TCAGTAGCTGCACTTACTAACAATGTTGCTTTTCAAACTGTCAAACCCGGTAATTT
TAACAAAGACTTCTATGACTTTGCTGTGTCTAAGGGTTTCTTTAAGGAAGGAAGTT
CTGTTGAATTAAAACACTTCTTCTTTGCTCAGGATGGTAATGCTGCTATCAGCGAT
TATGACTACTATCGTTATAATCTACCAACAATGTGTGATATCAGACAACTACTATTTG
TAGTTGAAGTTGTTGATAAGTACTTTGATTGTTACGATGGTGGCTGTATTAATGCTA
ACCAAGTCATCGTCAACAACCTAGACAAATCAGCTGGTTTTCCATTTAATAAATGG
GGTAAGGCTAGACTTTATTATGATTCAATGAGTTATGAGGATCAAGATGCACTTTT
CGCATATACAAAACGTAATGTCATCCCTACTATAACTCAAATGAATCTTAAGTATGC
CATTAGTGCAAAGAATAGAGCTCGCACCGTAGCTGGTGTCTCTATCTGTAGTACT
ATGACCAATAGACAGTTTCATCAAAAATTATTGAAATCAATAGCCGCCACTAGAGG
AGCTACTGTAGTAATTGGAACAAGCAAATTCTATGGTGGTTGGCACAACATGTTAA
AAACTGTTTATAGTGATGTAGAAAACCCTCACCTTATGGGTTGGGATTATCCTAAA
TGTGATAGAGCCATGCCTAACATGCTTAGAATTATGGCCTCACTTGTTCTTGCTCG
CAAACATACAACGTGTTGTAGCTTGTCACACCGTTTCTATAGATTAGCTAATGAGT
GTGCTCAAGTATTGAGTGAAATGGTCATGTGTGGCGGTTCACTATATGTTAAACC
AGGTGGAACCTCATCAGGAGATGCCACAACTGCTTATGCTAATAGTGTTTTTAAC
ATTTGTCAAGCTGTCACGGCCAATGTTAATGCACTTTTATCTACTGATGGTAACAA
AATTGCCGATAAGTATGTCCGCAATTTACAACACAGACTTTATGAGTGTCTCTATA
GAAATAGAGATGTTGACACAGACTTTGTGAATGAGTTTTACGCATATTTGCGTAAA
CATTTCTCAATGATGATACTCTCTGACGATGCTGTTGTGTGTTTCAATAGCACTTAT
GCATCTCAAGGTCTAGTGGCTAGCATAAAGAACTTTAAGTCAGTTCTTTATTATCA
AAACAATGTTTTTATGTCTGAAGCAAAATGTTGGACTGAGACTGACCTTACTAAAG
GACCTCATGAATTTTGCTCTCAACATACAATGCTAGTTAAACAGGGTGATGATTAT
GTGTACCTTCCTTACCCAGATCCATCAAGAATCCTAGGGGCCGGCTGTTTTGTAG
ATGATATCGTAAAAACAGATGGTACACTTATGATTGAACGGTTCGTGTCTTTAGCT
ATAGATGCTTACCCACTTACTAAACATCCTAATCAGGAGTATGCTGATGTCTTTCAT
TTGTACTTACAATACATAAGAAAGCTACATGATGAGTTAACAGGACACATGTTAGA
CATGTATTCTGTTATGCTTACTAATGATAACACTTCAAGGTATTGGGAACCTGAGTT
TTATGAGGCTATGTACACACCGCATACAGTCTTACAGGCTGTTGGGGCTTGTGTT
CTTTGCAATTCACAGACTTCATTAAGATGTGGTGCTTGCATACGTAGACCATTCTT

ATGTTGTAAATGCTGTTACGACCATGTCATATCAACATCACATAAATTAGTCTTGTC
TGTTAATCCGTATGTTTGCAATGCTCCAGGTTGTGATGTCACAGATGTGACTCAA
CTTTACTTAGGAGGTATGAGCTATTATTGTAAATCACATAAACCACCCATTAGTTTT
CCATTGTGTGCTAATGGACAAGTTTTTGGTTTATATAAAAATACATGTGTTGGTAGC
GATAATGTTACTGACTTTAATGCAATTGCAACATGTGACTGGACAAATGCTGGTGA
TTACATTTTAGCTAACACCTGTACTGAAAGACTCAAGCTTTTTGCAGCAGAAACG
CTCAAAGCTACTGAGGAGACATTTAAACTGTCTTATGGTATTGCTACTGTACGTGA
AGTGCTGTCTGACAGAGAATTACATCTTTCATGGGAAGTTGGTAAACCTAGACCA
CCACTTAACCGAAATTATGTCTTTACTGGTTATCGTGTAACTAAAAACAGTAAAGTA
CAAATAGGAGAGTACACCTTTGAAAAAGGTGACTATGGTGATGCTGTTGTTTACC
GAGGTACAACAACTTACAAATTAAATGTTGGTGATTATTTTGTGCTGACATCACATA
CAGTAATGCCATTAAGTGCACCTACACTAGTGCCACAAGAGCACTATGTTAGAATT
ACTGGCTTATACCCAACACTCAATATCTCAGATGAGTTTTCTAGCAATGTTGCAAA
TTATCAAAAGGTTGGTATGCAAAAGTATTCTACACTCCAGGGACCACCTGGTACT
GGTAAGAGTCATTTTGCTATTGGCCTAGCTCTCTACTACCCTTCTGCTCGCATAGT
GTATACAGCTTGCTCTCATGCCGCTGTTGATGCACTATGTGAGAAGGCATTAAAAT
ATTTGCCTATAGATAAATGTAGTAGAATTATACCTGCACGTGCTCGTGTAGAGTGTT
TTGATAAATTCAAAGTGAATTCAACATTAGAACAGTATGTCTTTTGTACTGTAAATG
CATTGCCTGAGACGACAGCAGATATAGTTGTCTTTGATGAAATTTCAATGGCCAC
AAATTATGATTTGAGTGTTGTCAATGCCAGATTACGTGCTAAGCACTATGTGTACA
TTGGCGACCCTGCTCAATTACCTGCACCACGCACATTGCTAACTAAGGGCACAC
TAGAACCAGAATATTTCAATTCAGTGTGTAGACTTATGAAAACTATAGGTCCAGAC
ATGTTCCTCGGAACTTGTCGGCGTTGTCCTGCTGAAATTGTTGACACTGTGAGT
GCTTTGGTTTATGATAATAAGCTTAAAGCACATAAAGACAAATCAGCTCAATGCTTT
AAAATGTTTTATAAGGGTGTTATCACGCATGATGTTTCATCTGCAATTAACAGGCC
ACAAATAGGCGTGGTAAGAGAATTCCTTACACGTAACCCTGCTTGGAGAAAAGCT
GTCTTTATTTCACCTTATAATTCACAGAATGCTGTAGCCTCAAAGATTTTGGGACTA
CCAACTCAAACTGTTGATTCATCACAGGGCTCAGAATATGACTATGTCATATTCAC
TCAAACCACTGAAACAGCTCACTCTTGTAATGTAAACAGATTTAATGTTGCTATTA
CCAGAGCAAAAGTAGGCATACTTTGCATAATGTCTGATAGAGACCTTTATGACAAG
TTGCAATTTACAAGTCTTGAAATTCCACGTAGGAATGTGGCAACTTTACAAGCTGA
AAATGTAACAGGACTTTTTAAAGATTGTAGTAAGGTAATCACTGGGTTACATCCTA
CACAGGCACCTACACACCTCAGTGTTGACACTAAATTCAAAACTGAAGGTTTATG
TGTTGACATACCTGGCATACCTAAGGACATGACCTATAGAAGACTCATCTCTATGA
TGGGTTTTAAAATGAATTATCAAGTTAATGGTTACCCTAACATGTTTATCACCCGCG
AAGAAGCTATAAGACATGTACGTGCATGGATTGGCTTCGATGTCGAGGGGTGTCA
TGCTACTAGAGAAGCTGTTGGTACCAATTTACCTTTACAGCTAGGTTTTTCTACAG
GTGTTAACCTAGTTGCTGTACCTACAGGTTATGTTGATACACCTAATAATACAGATT
TTTCCAGAGTTAGTGCTAAACCACCGCCTGGAGATCAATTTAAACACCTCATACC
ACTTATGTACAAAGGACTTCCTTGGAATGTAGTGCGTATAAAGATTGTACAAATGT
TAAGTGACACACTTAAAAATCTCTCTGACAGAGTCGTATTTGTCTTATGGGCACAT
GGCTTTGAGTTGACATCTATGAAGTATTTTGTGAAAATAGGACCTGAGCGCACCT
GTTGTCTATGTGATAGACGTGCCACATGCTTTTCCACTGCTTCAGACACTTATGC
CTGTTGGCATCATTCTATTGGATTTGATTACGTCTATAATCCGTTTATGATTGATGTT

CAACAATGGGGTTTTACAGGTAACCTACAAAGCAACCATGATCTGTATTGTCAAGT
CCATGGTAATGCACATGTAGCTAGTTGTGATGCAATCATGACTAGGTGTCTAGCT
GTCCACGAGTGCTTTGTTAAGCGTGTTGACTGGACTATTGAATATCCTATAATTGG
TGATGAACTGAAGATTAATGCGGCTTGTAGAAAGGTTCAACACATGGTTGTTAAA
GCTGCATTATTAGCAGACAAATTCCCAGTTCTTCACGCTATTGCAAACCCTAAAGC
TATTAAGTGTGTACCTCAAGCTGATGTAGAATGGAAGTTCTATGATGCACAGCCTT
GTAGTGACAAAGCTTATAAAATAGAAGAATTATTCTATTCTTATGCCACACATTCTG
ACAAATTCACAGATGGTGTATGCCTATTTTGGAATTGCAATGTCGATAGATATCCT
GCTAATTCCATTGTTTGTAGATTTGACACTAGAGTGCTATCTAACCTTAACTTGCCT
GGTTGTGATGGTGGCAGTTTGTATGTAAATAAACATGCATTCCACACACCAGCTTT
TGATAAAAGTGCTTTTGTTAATTTAAAACAATTACCATTTTTCTATTACTCTGACAGT
CCATGTGAGTCTCATGGAAAACAAGTAGTGTCAGATATAGATTATGTACCACTAAA
GTCTGCTACGTGTATAACACGTTGCAATTTAGGTGGTGCTGTCTGTAGACATCAT
GCTAATGAGTACAGATTGTATCTCGATGCTTATAACATGATGATCTCAGCTGGCTT
TAGCTTGTGGGTTTACAAACAATTTGATACTTATAACCTCTGGAACACTTTTACAA
GACTTCAGAGTTTAGAAAATGTGGCTTTTAATGTTGTAAATAAGGGACACTTTGAT
GGACAACAGGGTGAAGTACCAGTTTCTATCATTAATAACACTGTTTACACAAAAGT
TGATGGTGTTGATGTAGAATTGTTTGAAAATAAAACAACATTACCTGTTAATGTAGC
ATTTGAGCTTTGGGCTAAGCGCAACATTAAACCAGTACCAGAGGTGAAAATACTC
AATAATTTGGGTGTGGACATTGCTGCTAATACTGTGATCTGGGACTACAAAAGAG
ATGCTCCAGCACATATATCTACTATTGGTGTTTGTTCTATGACTGACATAGCCAAG
AAACCAACTGAAACGATTTGTGCACCACTCACTGTCTTTTTTGATGGTAGAGTTG
ATGGTCAAGTAGACTTATTTAGAAATGCCCGTAATGGTGTTCTTATTACAGAAGGT
AGTGTTAAAGGTTTACAACCATCTGTAGGTCCCAAACAAGCTAGTCTTAATGGAG
TCACATTAATTGGAGAAGCCGTAAAAACACAGTTCAATTATTATAAGAAAGTTGAT
GGTGTTGTCCAACAATTACCTGAAACTTACTTTACTCAGAGTAGAAATTTACAAGA
ATTTAAACCCAGGAGTCAAATGGAAATTGATTTCTTAGAATTAGCTATGGATGAATT
CATTGAACGGTATAAATTAGAAGGCTATGCCTTCGAA

- The sequences highlighted in yellow indicate unique MluI (ACGCGT) and BstBI (TTCGAA) restriction sites used to clone F4 (**Figure 1**).

Fragment 5 (F5)

TTCGAACATATCGTTTATGGAGATTTTAGTCATAGTCAGTTAGGTGGTTTACATCTA
CTGATTGGACTAGCTAAACGTTTTAAGGAATCACCTTTTGAATTAGAAGATTTTATT
CCTATGGACAGTACAGTTAAAAACTATTTCATAACAGATGCGCAAACAGGTTCATC
TAAGTGTGTGTGTTCTGTTATTGATTTATTACTTGATGATTTTGTTGAAATAATAAAA
TCCCAAGATTTATCTGTAGTTTCTAAGGTTGTCAAAGTGACTATTGACTATACAGA
AATTTCATTTATGCTTTGGTGTAAAGATGGCCATGTAGAAACATTTTACCCAAAATT
ACAATCTAGTCAAGCGTGGCAACCGGGTGTTGCTATGCCTAATCTTTACAAAATG
CAAAGAATGCTATTAGAAAAGTGTGACCTTCAAAATTATGGTGATAGTGCAACATT
ACCTAAAGGCATAATGATGAATGTCGCAAAATATACTCAACTGTGTCAATATTTAAA
CACATTAACATTAGCTGTACCCTATAATATGAGAGTTATACATTTTGGTGCTGGTTC
TGATAAAGGAGTTGCACCAGGTACAGCTGTTTTAAGACAGTGGTTGCCTACGGG
TACGCTGCTTGTCGATTCAGATCTTAATGACTTTGTCTCTGATGCAGATTCAACTT
TGATTGGTGATTGTGCAACTGTACATACAGCTAATAAATGGGATCTCATTATTAGTG
ATATGTACGACCCTAAGACTAAAAATGTTACAAAAGAAAATGACTCTAAAGAGGGT
TTTTTCACTTACATTTGTGGGTTTATACAACAAAAGCTAGCTCTTGGAGGTTCCGT
GGCTATAAAGATAACAGAACATTCTTGGAATGCTGATCTTTATAAGCTCATGGGAC
ACTTCGCATGGTGGACAGCCTTTGTTACTAATGTGAATGCGTCATCATCTGAAGC
ATTTTTAATTGGATGTAATTATCTTGGCAAACCACGCGAACAAATAGATGGTTATGT
CATGCATGCAAATTACATATTTTGGAGGAATACAAATCCAATTCAGTTGTCTTCCTA
TTCTTTATTTGACATGAGTAAATTTCCCCTTAAATTAAGGGGTACTGCTGTTATGTC
TTTAAAAGAAGGTCAAATCAATGATATGATTTTATCTCTTCTTAGTAAAGGTAGACT
TATAATTAGAGAAAACAACAGAGTTGTTATTTCTAGTGATGTTCTTGTTAACAACTA
AACGAACAATGTTTGTTTTTCTTGTTTTATTGCCACTAGTCTCTAGTCAGTGTGTTA
ATCTTACAACCAGAACTCAATTACCCCCTGCATACACTAATTCTTTCACACGTGGT
GTTTATTACCCTGACAAAGTTTTCAGATCCTCAGTTTTACATTCAACTCAGGACTT
GTTCTTACCTTTCTTTTCCAATGTTACTTGGTTCCATGCTATACATGTCTCTGGGA
CCAATGGTACTAAGAGGTTTGATAACCCTGTCCTACCATTTAATGATGGTGTTTATT
TTGCTTCCACTGAGAAGTCTAACATAATAAGAGGCTGGATTTTTGGTACTACTTTA
GACTCGAAGACCCAGTCCCTACTTATTGTTAATAACGCTACTAATGTTGTTATTAAA
GTCTGTGAATTTCAATTTTGTAATGATCCATTTTTGGGTGTTTATTACCACAAAAAC
AACAAAAGTTGGATGGAAAGTGAGTTCAGAGTTTATTCTAGTGCGAATAATTGCA
CTTTTGAATATGTCTCTCAGCCTTTTCTTATGGACCTTGAAGGAAAACAGGGTAAT
TTCAAAAATCTTAGGGAATTTGTGTTTAAGAATATTGATGGTTATTTTAAAATATATT
CTAAGCACACGCCTATTAATTTAGTGCGTGATCTCCCTCAGGGTTTTTCGGCTTTA
GAACCATTGGTAGATTTGCCAATAGGTATTAACATCACTAGGTTTCAAACTTTACTT
GCTTTACATAGAAGTTATTTGACTCCTGGTGATTCTTCTTCAGGTTGGACAGCTG
GTGCTGCAGCTTATTATGTGGGTTATCTTCAACCTAGGACTTTTCTATTAAAATATA
ATGAAAATGGAACCATTACAGATGCTGTAGACTGTGCACTTGACCCTCTCTCAGA
AACAAAGTGTACGTTGAAATCCTTCACTGTAGAAAAAGGAATCTATCAAACTTCTA
ACTTTAGAGTCCAACCAACAGAATCTATTGTTAGATTTCCTAATATTACAAACTTGT
GCCCTTTTGGTGAAGTTTTTAACGCCACCAGATTTGCATCTGTTTATGCTTGGAA
CAGGAAGAGAATCAGCAACTGTGTTGCTGATTATTCTGTCCTATATAATTCCGCAT

CATTTTCCACTTTTAAGTGTTATGGAGTGTCTCCTACTAAATTAAATGATCTCTGCT
TTACTAATGTCTATGCAGATTCATTTGTAATTAGAGGTGATGAAGTCAGACAAATC
GCTCCAGGGCAAACTGGAAAGATTGCTGATTATAATTATAAATTACCAGATGATTTT
ACAGGCTGCGTTATAGCTTGGAATTCTAACAATCTTGATTCTAAGGTTGGTGGTAA
TTATAATTACCTGTATAGATTGTTTAGGAAGTCTAATCTCAAACCTTTTGAGAGAGA
TATTTCAACTGAAATCTATCAGGCCGGTAGCACACCTTGTAATGGTGTTGAAGGTT
TTAATTGTTACTTTCCTTTACAATCATATGGTTTCCAACCCACTAATGGTGTTGGTT
ACCAACCATACAGAGTAGTAGTACTTTCTTTTGAACTTCTACATGCACCAGCAACT
GTTTGTGGACCTAAAAAGTCTACTAATTTGGTTAAAAACAAATGTGTCAATTTCAA
CTTCAATGGTTTAACAGGCACAGGTGTTCTTACTGAGTCTAACAAAAAGTTTCTG
CCTTTCCAACAATTTGGCAGAGACATTGCTGACACTACTGATGCTGTCCGTGATC
CACAGACACTTGAGATTCTTGACATTACACCATGTTCTTTTGGTGGTGTCAGTGT
TATAACACCAGGAACAAATACTTCTAACCAGGTTGCTGTTCTTTATCAGGATGTTA
ACTGCACAGAAGTCCCTGTTGCTATTCATGCAGATCAACTTACTCCTACTTGGCG
TGTTTATTCTACAGGTTCTAATGTTTTTCAAACACGTGCAGGCTGTTTAATAGGGG
CTGAACATGTCAACAACTCATATGAGTGTGACATACCCATTGGTGCAGGTATATGC
GCTAGTTATCAGACTCAGACTAATTCTCCTCGGCGGGCACGTAGTGTAGCTAGTC
AATCCATCATTGCCTACACTATGTCACTTGGTGCAGAAAATTCAGTTGCTTACTCT
AATAACTCTATTGCCATACCCACAAATTTTACTATTAGTGTTACCACAGAAATTCTA
CCAGTGTCTATGACCAAGACATCAGTAGATTGTACAATGTACATTTGTGGTGATTC
AACTGAATGCAGCAATCTTTTGTTGCAATATGGCAGTTTTTGTACACAATTAAACC
GTGCTTTAACTGGAATAGCTGTTGAACAAGACAAAAACACCCAAGAAGTTTTTGC
ACAAGTCAAACAAATTTACAAAACACCACCAATTAAAGATTTTGGTGGTTTTAATTT
TTCACAAATATTACCAGATCCATCAAAACCAAGCAAGAGGTCATTTATTGAAGATC
TACTTTTCAACAAAGTGACACTTGCAGATGCTGGCTTCATCAAACAATATGGTGAT
TGCCTTGGTGATATTGCTGCTAGAGACCTCATTTGTGCACAAAAGTTTAACGGCC
TTACTGTTTTGCCACCTTTGCTCACAGATGAAATGATTGCTCAATACACTTCTGCA
CTGTTAGCGGGTACAATCACTTCTGGTTGGACCTTTGGTGCAGGTGCTGCATTAC
AAATACCATTTGCTATGCAAATGGCTTATAGGTTTAATGGTATTGGAGTTACACAGA
ATGTTCTCTATGAGAACCAAAAATTGATTGCCAACCAATTTAATAGTGCTATTGGCA
AAATTCAAGACTCACTTTCTTCCACAGCAAGTGCACTTGGAAAACTTCAAGATGT
GGTCAACCAAAATGCACAAGCTTTAAACACGCTTGTTAAACAACTTAGCTCCAATT
TTGGTGCAATTTCAAGTGTTTTAAATGATATCCTTTCACGTCTTGACAAAGTTGAG
GCTGAAGTGCAAATTGATAGGTTGATCACAGGCAGACTTCAAAGTTTGCAGACAT
ATGTGACTCAACAATTAATTAGAGCTGCAGAAATCAGAGCTTCTGCTAATCTTGCT
GCTACTAAAATGTCAGAGTGTGTACTTGGACAATCAAAAAGAGTTGATTTTTGTG
GAAAGGGCTATCATCTTATGTCCTTCCCTCAGTCAGCACCTCATGGTGTAGTCTT
CTTGCATGTGACTTATGTCCCTGCACAAGAAAAGAACTTCACAACTGCTCCTGCC
ATTTGTCATGATGGAAAAGCACACTTTCCTCGTGAAGGTGTCTTTGTTTCAAATG
GCACACACTGGTTTGTAACACAAAGGAATTTTTATGAACCACAAATCATTACTACA
GACAACACATTTGTGTCTGGTAACTGTGATGTTGTAATAGGAATTGTCAACAACAC
AGTTTATGATCCTTTGCAACCTGAATTAGACTCATTCAAGGAGGAGTTAGATAAAT
ATTTTAAGAATCATACATCACCAGATGTTGATTTAGGTGACATCTCTGGCATTAATG
CTTCAGTTGTAAACATTCAAAAAGAAATTGACCGCCTCAATGAGGTTGCCAAGAA

TTTAAATGAATCTCTCATCGATCTCCAAGAACTTGGAAAGTATGAGCAGTATATAAA ATGGCCATGGTACATTTGGCTAGGTTTTATAGCTGGCTTGATTGCCATAGTAATGG TGACAATTATGCTTTGCTGTATGACCAGTTGCTGTAGTTGTCTCAAGGGCTGTTG TTCTTGTGGATCC

- Sequences highlighted in yellow represent unique BstBI (TTCGAA) and BamHI (GGATCC) restriction sites used to clone F5 (**Figure 1**).

- The single nucleotide highlighted in green represents a silent mutation introduced to remove a BstBI (TTCGAA) restriction site present in the viral spike (S) gene to clone F5 (**Figure 1**). This single nucleotide silent mutation was also used as a genetic tag to distinguish the rSARS-CoV-2 from the natural SARS-CoV-2 isolate.

Venus-2A

ATGGTGAGCAAGGGCGAGGAGCTGTTCACCGGGGTGGTGCCCATCCTGGTCGA
GCTGGACGGCGACGTAAACGGCCACAAGTTCAGCGTGTCCGGCGAGGGCGAG
GGCGATGCCACCTACGGCAAGCTGACCCTGAAGCTGATCTGCACCACCGGCAA
GCTGCCCGTGCCCTGGCCCACCCTCGTGACCACCCTGGGCTACGGCCTGCAG
TGCTTCGCCCGCTACCCCGACCACATGAAGCAGCACGACTTCTTCAAGTCCGCC
ATGCCCGAAGGCTACGTCCAGGAGCGCACCATCTTCTTCAAGGACGACGGCAA
CTACAAGACCCGCGCCGAGGTGAAGTTCGAGGGCGACACCCTGGTGAACCGC
ATCGAGCTGAAGGGCATCGACTTCAAGGAGGACGGCAACATCCTGGGGCACAA
GCTGGAGTACAACTACAACAGCCACAACGTCTATATCACCGCCGACAAGCAGAA
GAACGGCATCAAGGCCAACTTCAAGATCCGCCACAACATCGAGGACGGCGGCG
TGCAGCTCGCCGACCACTACCAGCAGAACACCCCCATCGGCGACGGCCCCGTG
CTGCTGCCCGACAACCACTACCTGAGCTACCAGTCCAAGCTGAGCAAAGACCC
CAACGAGAAGCGCGATCACATGGTCCTGCTGGAGTTCGTGACCGCCGCCGGGA
TCACTCTCGGCATGGACGAGCTGTACAAAGGGTCCGGAGCCACGAACTTCTCTC
TGTTAAAGCAAGCAGGGGACGTGGAAGAAAACCCCGGTCCT

- The underlined sequence indicates the porcine Teschovirus-1 (PTV-1) 2A self-cleaving peptide.
